# Genetic variation and characterization of Bambara groundnut [*Vigna subterranea* (L.) verdc.] accessions under multi-environments considering yield and yield components performance

**DOI:** 10.1038/s41598-023-28794-8

**Published:** 2023-01-27

**Authors:** Vincent Ishola Esan, Grace Oluwasikemi Oke, Timothy Oyebamiji Ogunbode

**Affiliations:** Environmental Management and Crop Production Unit, B. Agriculture Program, College of Agriculture, Engineering and Science, Bowen University, Iwo, Nigeria

**Keywords:** Genetics, Plant sciences, Environmental sciences

## Abstract

Bambara groundnut has significant role to play in terms of food security, even though researchers in agriculture have paid very little attention to the crop in the past. This study aimed to investigate the high-yielding accessions in three environments. A total of 34 phenological, vegetative and yield traits were measured and analyzed statistically with R software. There were significant differences in all the traits except for plant height, initial plant stand, panicle length per stem, and petiole length. Across the three environments, TVSU-455 gave the highest values for the total number of pods (42.67), final plant stands (7.67), fresh seed weights (45.83), number of seeds per plant (46.62), hundred seed weight with a value (124.56), dry seed weight (27.14), fresh pod weight (92.65), harvest index of 0.57, yield per plot (45.83) and unshelled yield per plot (550.26). TVSU-455 was the only accession in cluster I of the dendrogram based on its superiority over other accessions. The clustering analysis produced a dendrogram categorizing the 15 accessions into 4 groups based on the vegetative, phenological, and yield traits. There were significant differences among the correlations of the 34 traits. The first two principle components explained 56.16% of the total variation with each dimension accounting for 39.85% and 16. 31% variation, respectively. TVSU-455 can be recommended for stability analysis.

## Introduction

Bambara groundnut (*Vigna subterranea L. Verdc*) is an annual legume belonging to the family of *Fabaceae*,* a* subfamily of Faboidea and genus Vigna^1^. The seed qualities, pod, color, texture, leaf shape, plant vigor, and nutritional and anti-nutritional qualities of the crop differ from one variety to another. It is said to have originated from West Africa (Chad, Central African Republic, Nigeria, and Cameroon). This crop can also be found in some tropical parts like America, Australia, and Asia but the level of its cultivation is very low^2^.

Bambara groundnut ranks third as the most common essential crop behind peanuts *(Arachis hypogea)* and cowpeas *(Vigna unguiculata)* in Africa but is not classified globally in the world’s trading scheme because it is underutilized and researchers have paid very little attention to the crop because its rank in the production percentages was low^3^. This crop is said to be underutilized and often regarded as a “poor man’s crop” and/or “women’s crop” simply because all the pre-field, field, and post-field activities are often performed by women^4^ and also being grown at the subsistence level to meet the family’s immediate needs^1^. However, it was recently noticed and declared as a crop for the new millennium due to its characteristic richness in nutrients and its resilient to climate change^5^.

It can be used in different ways or processed into diverse food products by different people and cultures across different locations where it is grown. The recorded setbacks to the use of the bambara seeds are in its hard-to-cook and hard-to-mill challenges which often lead to an extended cooking time before the adequate softening texture for consumption is achieved^6^. Also contributing to the low adoption of Bambara by many farmers are the lack of practicing modern production techniques and the fact that breeders still have to use the local variety or natural breed of the crop^7^.

The green freshly harvested groundnuts can be cooked with spice and salt, often consumed by West Africans^8^. Although Mazahib et al^9^ reported that the methods to shorten the length of cooking of the crop has been a source of major concern for researchers and farmers, the results of a survey revealed that the seeds could be roasted or soaked as a pretreatment means before milling into flour to combat the hard-to-cook problem^10^. Also, in previous surveys, soaking and roasting of the seeds has been employed to bypass the hard-to-mill challenge^11^. Bambara groundnuts have been reportedly found to be highly nutritious compared to other legumes and are the preferred food crop of many local people and people who cannot afford to buy valuable animal protein products^12^.

Bambara groundnut can be processed into many end products after harvesting using different methods^13–16^, it can be used by adults or babies as food supplement^17^ because it has a higher rate of acceptance than soybean and cowpea milk^18^. Many parts of Bambara plants are used as animal feed^19^ in the tropics. It is very rich in protein contributing up to 25% of world protein^20^. In Congo, oil is extracted from Bambara seeds^21^.

Aside from its nutritional benefits, Bambara also has diverse health benefits which range from being used to cure diarrhea^22^, prevent high blood pressure^23^, treat wounds and heal epilepsy^20^, and control vomiting during pregnancy when chewed and swallowed^24^.

Because Bambara nut is a leguminous crop highly rich in nutrients, it is referred to as “a complete balanced diet “due to its high carbohydrate content (49–64.5%), protein (15–25.5%), fat (4.5–7.4%), fiber (5.2–6.4%), ash (3.2–4.4%), minerals (2%) and it also contains micronutrients such as K (11.45–19.35 mg/100 g), Fe (5.1–9.2 mg/100 g), Na (2.9–12.0 mg/100 g), and Ca (95.8–99 mg/100 g) present in its seed^25^. The biochemical investigation that was also carried out revealed that Bambara groundnut contains nonessential amino acids of (66.69%) and essential amino acids of (33.31%)^26^. Like other leguminous crops, it can help fix the atmospheric nitrogen in the soil^27^.

Bambara groundnut is drought-resistant and it can thrive well in area of nutrient-poor soil, even where some other crops fail^28^. It can safeguard future food and dietary needs in face of climate change due to the crop’s intrinsic resistance to stress^29^. To small-scale or subsistence farmers in situations of low agricultural inputs like fertilizers and pesticides for production, Bambara groundnut can still thrive well. The roots of Bambara groundnut help to fix nitrogen in the soil, thereby replenishing and repairing the nutrients in the soil. This attribute makes it highly suitable to be rotated or intercropped with rice, maize, millet, yam, or cassava to mention few^20^. The leaves of the crop are rich in nitrogen and potassium which also renders it suitable for animal feed.

Annual world Bambara groundnut production is estimated at approximately 0.3 million tons, of which 0.2 million tons were produced in Africa^30^. The largest producers of Bambara groundnut in Africa are Burkina Faso, Niger, and Cameroon and Nigeria^30^. Nigeria leads gross Bambara groundnut production with 100,000 metric tons per annum while Burkina Faso leads with highest production yield^8^. It should be noted that the existing natural breeds produced low yields due to abiotic and biotic stress such as diseases and pests’ outbreaks and a lack of genotype improvements to adapt to climate change^28^^,^^31,70^. On the other hand, according to Azam-Ali^13^, some researchers have noted that improved Bambara groundnut genotypes can produce yield from 3.0 to 4.5 t/ha when all factors related to yield are correlated. It is also noteworthy that some factors such as rainfall, temperature, humidity, agro-pedology, and abiotic/biotic cause differences in agronomic performance, especially yield stability and yield quality of the crops as a way of responding to different environment^33,34^. It is important to study vegetative, phenological, and yield traits under multi-environments to select the best traits contributing to stability and high yields of genotypes, which can be recommended to farmers and made available to breeders as breeding lines for Bambara improvement in different breeding programs. The morphological and phenological performance is of paramount importance to high potential agronomic outcomes, which the current investigation assessed. Therefore, the objectives of this study were to (i) determine the phenological, vegetative, yield traits, and qualitative trait performances of 15 Bambara groundnut accessions across environments of three different climatic scenarios.

## Results

### Soil analysis

Higher amount of sand, bray P, %OC, Mn, Na, and Mg were recorded in Bowen, higher amount of clay and Cu were found in Ibadan while higher amount of silt, %N, Zn, Fe, K and Ca were found in Odeda (Table 1). The pH at Bowen, Ibadan, and Odeda were 7.20, 7.64, and 6.79, respectively.

**Table 1:** The physio-chemical properties of soil for the 3 experimental locations.

Properties	Bowen	Ibadan	Odeda
Sand %	71.00	60.22	56.22
Clay %	10.90	29.14	13.63
Silt %	18.10	10.64	30.15
% N	0.19	0.20	1.12
Bray P	23.11	14.84	17.26
% OC	1.19	0.99	0.84
Zn (ppm)	1.38	1.30	2.30
Cu (ppm)	0.68	1.20	0.98
Fe (ppm)	77.96	82.40	89.62
Mn (ppm)	169.42	108.17	117.64
Na (Cmol/kg)	0.09	0.03	0.08
K (Cmol/kg)	0.43	0.27	1.23
Mg (Cmol/kg)	2.50	2.00	0.75
Ca (Cmol/kg)	4.75	2.78	5.13
pH	7.20	7.64	6.79

### Characterization of Bambara groundnut accessions using morphological, phenological, and agronomic traits

The ANOVA for the morphological is presented in Table 2. The least internode length (2.49 ± 0.63 cm) was recorded with TVSU-158 while TVSU-194 had the longest internode length (2.95 ± 0.64) across the environments, with a grand mean value of 2.69 cm. The Leaf length ranged from 6.96 ± 1.44 (TVSU-1520) to 8.47 ± 1.53 (TVSU-194), while the minimum value across environments was 3.50 cm and the maximum length was 12.60 cm, with a grand mean value of 7.53 cm. The Leaf width was recorded with the lowest value of 2.83 ± 0.55 and 2.83 ± 0.60 in TVSU-513 and TVSU-2096, respectively, and the highest width (3.27 ± 0.71) was recorded in TVSU-438, with a grand mean was 2.98 cm. Additionally, the plant height ranged from 24.41 ± 3.14 (TVSU-643) to 29.53 ± 4.19 (TVSU-939) and a grand mean value of 27.12 cm across environments. TVSU-1920 was observed to show the lowest initial plant stand with a value of 4.67 ± 2.06 while TVSU-455 showed the most initial plant with a grand mean of 6.02. The values for the number of branches varied from 11.85 ± 5.00 (TVSU-939) to 20.24 ± 4.89 (TVSU-1611) although, the maximum across environments was 35.00 while the minimum was 5.00. For the number of nodes, TVSU-1920 performed least while TVSU-455 performed best with means of 9.67 ± 2.78 and 12.87 ± 2.84, respectively. Also, TVSU-1392 gave the least panicle length of 2.38 ± 0.70 while the longest panicle length (2.77 ± 0.80 cm) was observed in TVSU-633. The biomass fresh weight ranged from 53.64 ± 22.97 (V3) to 74.32 ± 34.47 (TVSU-1531) with a grand mean of 64.41, while the biomass dry weight varied from 25.85 ± 9.90 (TVSU-633) to 33.20 ± 12.15 (TVSU-2096). TVSU-1920 recorded the least biomass per plant of 44.33 ± 8.23 while the most biomass per plant of 50.89 ± 8.39 was found in TVSU-643. The least number of stems was observed in TVSU-643, while TVSU-455 produced the highest number of stems which ranged from 60.29 ± 15.56 to 79.67 ± 21.68. Two different varieties TVSU-158 and TVSU-1520 showed an equal number of leaves (204.05 and 204.05) but the least number of leaves was found in TVSU-643 (179.37 ± 47.51) while the highest was recorded in TVSU-455 (236.93 ± 65.21). Lastly, the length of petioles ranged between 1.66 ± 0.38 (TVSU-454) and 1.87 ± 0.42 (V4) with a grand mean of 1.77 cm.

**Table 2:** ANOVA for vegetative parameters after 82 days of planting.

Variety	IL50%	LL50%	LW50%	PH50%	IPS50%	BFW	BDW	BPP	LP50%	NL50%	NS50%
TVSU-454	2.64 ± 0.43bcd	7.69 ± 1.18bcde	3.14 ± 0.56abc	29.29 ± 4.03ab	5.56 ± 2.46	69.90 ± 24.39abc	33.00 ± 11.57abc	45.11 ± 9.72abcd	1.66 ± 0.38	233.42 ± 64.88abc	74.24 ± 21.62abcd
TVSU-158	2.49 ± 0.63d	7.54 ± 1.09bcdef	2.88 ± 0.42defg	26.41 ± 3.24bc	5.89 ± 2.15	55.43 ± 24.46de	26.61 ± 12.19f.	48.24 ± 9.33abcd	1.79 ± 0.36	204.05 ± 51.87 cd	68.83 ± 15.45de
TVSU-438	2.54 ± 0.64d	7.70 ± 1.34bcd	3.27 ± 0.71a	25.85 ± .63bc	6.78 ± 2.44	53.64 ± 22.97e	26.16 ± 11.02f.	48.97 ± 7.40abc	1.80 ± 0.44	185.32 ± 52.20de	61.77 ± 17.40ef
TVSU-633	2.84 ± 0.74ab	7.30 ± 1.32cdefg	2.99 ± 0.39cde	25.56 ± 3.69bc	5.11 ± 1.96	58.33 ± 22.09de	25.85 ± 9.90f.	45.15 ± 10.16bcd	1.87 ± 0.42	208.54 ± 42.83bc	69.51 ± 14.28 cd
TVSU-1520	2.53 ± 0.53d	6.96 ± 1.44 g	2.89 ± 0.53cdefg	25.48 ± 4.39bc	5.25 ± 1.16	73.69 ± 31.55ab	32.81 ± 11.52abcd	46.77 ± 10.90abcd	1.69 ± 0.39	204.16 ± 57.76 cd	68.05 ± 19.25de
TVSU-939	2.61 ± 0.65bcd	7.81 ± 1.13bd	2.98 ± 0.56cdef	29.28 ± 4.19ab	6.11 ± 2.80	77.23 ± 35.69a	31.70 ± 10.80abcde	44.41 ± 12.40d	1.77 ± 0.46	234.68 ± 60.26a	76.50 ± 18.27abc
TVSU-513	2.52 ± 0.58d	7.07 ± 1.12 fg	2.83 ± 0.55efg	25.86 ± 2.61bc	6.11 ± 1.27	56.48 ± 21.86de	27.81 ± 11.88f.	49.21 ± 7.43ab	1.73 ± 0.41	218.11 ± 56.27abc	72.77 ± 18.85abcd
TVSU-455	2.91 ± 0.63a	7.96 ± 1.29b	3.24 ± 0.67ab	29.04 ± 4.21ab	7.67 ± 2.45	71.78 ± 21.17abc	35.21 ± 11.56a	49.18 ± 8.09ab	1.70 ± 0.43	236.93 ± 65.21a	79.67 ± 21.68a
TVSU-643	2.63 ± 0.64bcd	7.28 ± 1.02defg	2.74 ± 0.56 fg	24.41 ± 3.14c	5.78 ± 2.17	56.07 ± 20.38de	27.98 ± 9.43def	50.89 ± 8.39a	1.76 ± 0.39	179.37 ± 47.51e	60.29 ± 15.56f.
TVSU-2096	2.79 ± 0.52abc	7.35 ± 1.10cdefg	2.83 ± 0.60efg	26.73 ± 2.84bc	6.56 ± 2.79	65.41 ± 21.47bcd	33.20 ± 12.15ab	50.63 ± 8.55a	1.84 ± 0.38	222.23 ± 44.09abc	74.08 ± 14.70abcd
TVSU-194	2.95 ± 0.64a	8.47 ± 1.53a	3.10 ± 0.64abcd	32.96 ± 3.88a	7.44 ± 2.35	65.07 ± 19.89bcd	31.82 ± 10.17abcde	50.21 ± 11.39a	1.75 ± 0.44	216.64 ± 45.16abc	72.16 ± 15.22bcd
TVSU-1611	2.55 ± 0.37 cd	7.19 ± 0.92efg	3.05 ± 0.61abcde	25.81 ± 3.87bc	5.78 ± 2.22	62.40 ± 19.43cde	29.10 ± 10.28bcdef	49.26 ± 11.79ab	1.73 ± 0.30	207.29 ± 41.14 cd	69.10 ± 13.71cde
TVSU-1920	2.86 ± 0.58ab	7.36 ± 0.66cdefg	3.01 ± 0.58bcde	27.77 ± 3.27bc	4.67 ± 2.06	72.79 ± 25.99abc	31.63 ± 10.13abcde	44.33 ± 8.23d	1.83 ± 0.44	231.53 ± 59.93ab	77.64 ± 19.10ab
TVSU-1531	2.85 ± 0.59ab	7.72 ± 1.26bcd	3.04 ± 0.58abcde	28.90 ± 3.43ab	5.89 ± 2.15	74.32 ± 34.47ab	32.40 ± 12.92abcde	45.04 ± 8.83 cd	1.83 ± 0.47	225.83 ± 50.33abc	75.22 ± 16.74abcd
TVSU-1392	2.59 ± 0.51 cd	7.32 ± 0.96cdefg	2.70 ± 0.45 g	24.41 ± 3.29c	5.67 ± 2.91	56.55 ± 20.60de	28.24 ± 9.99cdef	49.45 ± 7.82a	1.72 ± 0.46	206.71 ± 46.59 cd	68.21 ± 15.48de
MIN	1.10	3.50	1.60	14.40	1.00	16.40	5.80	22.06	1.00	47.00	19.00
MAX	5.50	12.60	5.40	40.80	11.00	181.80	65.10	83.56	3.20	396.00	127.00
Grand means	2.69	7.53	2.98	27.12	6.02	64.41	30.27	48.07	1.77	213.52	71.16
CV	21.48	15.43	19.00	14.36	33.22	38.38	36.57	19.67	23.32	24.68	24.23
MS	3.795***	6.787***	1.379***	235.1**	6.80^ ns^	4008***	394.6**	29,929***	1.398^ ns^	12,045***	1266.9***
LSD	0.690	0.936	0.605	5.418	-	42.592	12.035	13.663	-	76.828	25.606

IL50%: Internode Length (cm), IPS50%: Initial plant stand, LL50%: Leaf length (cm), LW50%: Leaf width (cm), PH50%: Plant height (cm), NL50%: Number of leaves per plant, NS: Number of stem per plant, LP50%: Petiole length per stem (cm), Min: Minimum across environment, Max: Maximum across environment, *Significant at *p* ≤ 0.05; **highly significant at *p* ≤ 0.01; ns = non-significant *p* > 0.05 and very highly significant at ***, ns: not significant. *CV* coefficient of variation, *BFW* biomass fresh weight per plant (g), *BDW* biomass dry weight per plant (g), *BPP* biomass per plant (BDW/BFW × 100). *MS* mean square, *LSD* least significant difference.

The phonological traits showed very highly significant differences between the accessions subjected to the experiment. The coefficient of variation (CV%) in this present research ranged from 4.22 to 8.60% while the Pr(< F) ranged from 7.44e-14*** to < 2e-16***which indicates a very highly significant difference (*p* ≤ 0.0001) and this was observed in days to emergence, days to flowering, days to maturity and days to harvest (Table 3).

**Table 3:** ANOVA for phenological traits and vegetative traits (continued).

Variety	DTE	DTF	DTM	DTH	NB	NN	PL
TVSU-454	7.44 ± 0.73ef	36.67 ± 4.09ghi	92.44 ± 1.67hi	105.44 ± 5.46 fg	12.97 ± 6.93 g	12.05 ± 2.88abc	2.67 ± 0.44
TVSU-158	8.22 ± 0.44d	42.56 ± 3.64cde	93.67 ± 1.66gh	108.78 ± 5.97def	17.39 ± 3.65bcdef	10.32 ± 2.05fgh	2.50 ± 0.57
TVSU-438	7.33 ± 0.50f.	39.78 ± 1.79efg	91.44 ± 2.16hi	104.78 ± 6.57 fg	19.34 ± 3.90ab	11.59 ± 2.20bcde	2.71 ± 0.58
TVSU-633	8.00 ± 0.00de	43.00 ± 3.54cde	101.89 ± 6.49de	110.33 ± 4.09cde	16.38 ± 5.19ef	10.49 ± 2.99efgh	2.77 ± 0.80
TVSU-1520	9.00 ± 1.22c	53.67 ± 4.80a	117.78 ± 1.64a	120.67 ± 2.50a	18.87 ± 3.97abc	10.82 ± 2.99defgh	2.67 ± 0.71
TVSU-939	7.44 ± 0.53ef	35.89 ± 3.06hi	89.56 ± 2.13i	103.33 ± 4.21 g	11.85 ± 5.00 g	11.38 ± 2.00bcdef	2.70 ± 0.60
TVSU-513	11.44 ± 0.73a	43.22 ± 3.77 cd	109.67 ± 5.10b	112.56 ± 4.98 cd	18.84 ± 3.72abc	10.18 ± 2.50gh	2.47 ± 0.83
TVSU-455	10.67 ± 0.50b	48.33 ± 2.60b	114.33 ± 2.40a	119.44 ± 2.51ab	17.82 ± 3.98bcde	12.87 ± 2.84a	2.67 ± 0.60
TVSU-643	8.11 ± 0.33d	36.00 ± 2.29hi	97.00 ± 7.89 fg	106.33 ± 8.79efg	18.29 ± 3.89abcde	11.98 ± 2.51abc	2.60 ± 053
TVSU-2096	8.89 ± 0.33c	39.89 ± 4.81efg	92.22 ± 3.90hi	103.44 ± 4.56 g	16.23 ± 6.87def	10.95 ± 3.26cdefg	2.58 ± 0.69
TVSU-194	8.89 ± 1.05c	45.56 ± 4.75bc	108.56 ± 7.33b	115.00 ± 5.68bc	15.71 ± 3.84f.	11.84 ± 3.05abcd	2.40 ± 0.51
TVSU-1611	8.00 ± 0.00de	34.56 ± 1.67i	101.22 ± 4.76de	108.78 ± 4.79def	20.24 ± 4.89a	11.63 ± 1.87bcde	2.62 ± 0.46
TVSU-1920	9.11 ± 0.33c	37.89 ± 4.34fgh	106.56 ± 4.28bc	113.56 ± 4.98 cd	17.17 ± 5.15cdef	9.67 ± 2.78 h	2.56 ± 0.58
TVSU-1531	7.44 ± 0.53ef	42.89 ± 3.82cde	98.00 ± 2.65ef	110.78 ± 4.94de	17.07 ± 4.53cdef	11.12 ± 2.85bcdefg	2.48 ± 0.47
TVSU-1392	11.78 ± 0.67a	41.11 ± 2.09def	103.89 ± 2.85 cd	113.11 ± 4.88 cd	18.64 ± 4.89abcd	12.24 ± 3.03ab	2.38 ± 0.70
MIN	7.00	30.00	86.00	95.00	5.00	4.00	1.20
MAX	12.00	60.00	120.00	125.00	35.00	19.00	5.60
Grand means	8.79	41.40	101.21	110.4	17.19	11.30	2.58
CV	7.04	8.60	4.22	4.73	27.63	23.14	23.67
MS	8.086***	95.57***	263.9***	97.37***	107.85***	11.35***	0.289 ns
LSD	7.581	1.192	1.029	2.893	9.763	5.642	1.762

*DTE* days to emergence, *DTF* days to flowering, *DTM* days to maturity, *DTH* days to harvest, *NB* number of branches per plant, *PL* panicle length per stem (cm), *NN* number of nodes per stem. *MS* mean square, *LSD* least significant difference.

The yield and yield component traits are shown in Table 4. There were very highly significant differences among all the means recorded. In ten out of the 17 agronomic traits, TVSU-455 was observed and recorded to be the best genotype across the three environments. TVSU-455 gave the highest values for the total number of pods (42.67 ± 13.37), final plant stand (7.67 ± 1.58), fresh seed weight (45.83 ± 14.82), number of seeds per plant (46.62 ± 14.89), hundred seed weight (124.56 ± 18.99), dry seed weight (27.14 ± 8.91), fresh pod weight (92.65 ± 30.96), harvest index (0.57 ± 0.25), yield per plot (45.83 ± 14.82) and unshelled yield per plot (550.26 ± 117.89). Additionally, it was observed that TVSU-455 gave higher values than the grand means for those 10 traits that it performed best at. Although, TVSU-1531 recorded 100 pods in a single environment i.e. Bowen. In the alternative, TVSU-1520 gave the least values for the traits which include final plant stand (3.77 ± 1.99), yield per plant (20.25 ± 10.87), hundred seed weight (76.15 ± 16.58), yield per plot (95.33 ± 52.95), dry seed weight (11.57 ± 5.54), the width of seed (9.12 ± 1.65) and harvest index (0.25 ± 0.2). Also, TVSU-1531 gave the least values for fresh pod weight (46.66 ± 23.13), length of pod (17.56 ± 2.82), width of pod (12.87 ± 2.33) and unshelled yield per plot (220.10 ± 87.20). TVSU-1392 gave the lowest value for final plant stand (3.77 ± 1.99). TVSU-454 gave the least value for number of seeds per plant (26.08 ± 7.40). TVSU-194 gave the lowest value for shelling percentage (41.91 ± 12.98) and it gave the highest value for the width of pods (16.29 ± 2.16). TVSU-158 gave the highest value for length of pods (23.40 ± 3.53). TVSU-633 gave the highest value for the width of seed (11.29 ± 2.26). TVSU-1920 also reported the highest value in length of seed (14.02 ± 1.64) and TVSU-939 was observed to perform best in shelling percentage (52.78 ± 14.17).

**Table 4:** ANOVA for yields and yield components.

Variety	TNP	FPS	FSW	NSP	HSW	YPL	DSW		
TVSU-454	26.22 ± 6.86e	4.86 ± 2.10bcde	25.14 ± 6.40efgh	26.08 ± 7.40f.	95.41 ± 8.94cdef	117.56 ± 43.43def	14.61 ± 3.89fgh		
TVSU-158	28.32 ± 10.52de	5.33 ± 1.94bcd	28.74 ± 10.64def	30.15 ± 9.97cdef	102.14 ± 15.45bcd	152.77 ± 77.44def	17.78 ± 7.00ef		
TVSU-438	34.09 ± 10.72bc	6.33 ± 1.58ab	38.63 ± 11.41b	37.05 ± 10.32b	102.27 ± 8.65bcd	226.91 ± 62.62b	22.97 ± 6.93b		
TVSU-633	26.41 ± 11.10e	4.44 ± 1.67de	31.62 ± 11.86 cd	28.81 ± 11.34cdef	111.71 ± 21.40ab	141.21 ± 63.06def	18.38 ± 7.26de		
TVSU-1520	26.26 ± 8.88e	4.63 ± 1.19cde	20.25 ± 10.87 h	26.63 ± 8.89ef	76.15 ± 16.58 g	95.33 ± 52.95f.	11.57 ± 5.54 h		
TVSU-939	35.25 ± 12.24b	5.33 ± 1.80bcd	31.04 ± 9.97 cd	33.58 ± 11.57bc	101.52 ± 15.37bcd	158.63 ± 53.81cde	18.95 ± 6.45cde		
TVSU-513	27.91 ± 8.50de	5.56 ± 1.13bcd	25.02 ± 9.67fgh	29.33 ± 8.46cdef	98.78 ± 8.21bcde	139.51 ± 44.80def	15.23 ± 6.13 fg		
TVSU-455	42.67 ± 13.37a	7.67 ± 1.58a	45.83 ± 14.82a	46.62 ± 14.89a	124.56 ± 18.89a	319.51 ± 44.80a	27.14 ± 8.91a		
TVSU-643	25.37 ± 8.97e	4.78 ± 1.79cde	25.27 ± 10.41efgh	26.74 ± 8.83ef	97.09 ± 16.62cdef	120.76 ± 65.53def	15.07 ± 5.62 fg		
TVSU-2096	29.59 ± 13.45cde	6.30 ± 2.75ab	34.93 ± 17.02bc	31.12 ± 13.90cde	104.43 ± 16.14bc	208.41 ± 116.67bc	20.85 ± 10.38bcd		
TVSU-194	28.14 ± 9.98de	6.00 ± 2.00bc	29.97 ± 13.16de	28.75 ± 12.27def	103.01 ± 15.84bcd	175.58 ± 91.31bcd	18.35 ± 7.80de		
TVSU-1611	26.95 ± 10.61de	4.67 ± 1.80cde	23.29 ± 10.12gh	29.05 ± 10.87cdef	90.40 ± 7.80defg	116.10 ± 65.79ef	14.07 ± 6.32gh		
TVSU-1920	31.58 ± 8.24 cd	4.56 ± 1.94cde	35.10 ± 9.96bc	32.26 ± 8.93bcd	105.26 ± 18.94bc	159.84 ± 79.31cde	22.03 ± 6.69bc		
TVSU-1531	29.20 ± 16.29de	5.33 ± 2.00bcd	21.38 ± 11.16 h	30.98 ± 14.70cdef	83.60 ± 13.50 fg	107.87 ± 37.86ef	12.96 ± 6.66gh		
TVSU-1392	28.44 ± 9.26e	3.77 ± 1.99e	28.51 ± 10.42defg	29.91 ± 9.70cdef	86.39 ± 13.10efg	105.11 ± 61.55ef	17.84 ± 6.04def		
MIN	5.00	1.00	1.80	2.00	50.20	11.2	0.90		
MAX	100.00	5.32	85.50	97.00	163.40	408.90	55.00		
Grand means	29.96	11.00	29.93	31.38	99.10	157.50	18.02		
CV	36.32	30.46	38.29	35.38	15.20	38.74	38.56		
MS	195.73***	15.786***	1984.8***	1149.5***	1178.8***	30,038***	719.4***		
LSD	15.81	3.029	18.303	18.324	29.24	156.521	10.942		

Variety	FPW	MPN	LOP	WOP	LOS	WOS	SP	HI	YPU
TVSU-454	59.54 ± 15.42ef	24.57 ± 6.91de	20.19 ± 3.60def	14.32 ± 2.20cde	12.45 ± 1.78 fg	9.64 ± 1.21fgh	42.55 ± 4.62ef	0.31 ± 0.09ef	252.66 ± 93.42de
TVSU-158	59.61 ± 26.13ef	26.88 ± 10.58de	23.40 ± 3.53a	14.23 ± 2.04de	13.07 ± 1.35cdef	10.15 ± 1.88bcdef	51.29 ± 12.87ab	0.39 ± 0.22d	284.24 ± 136.85cde
TVSU-438	77.54 ± 24.04b	31.66 ± 10.68bc	22.16 ± 3.95abc	14.28 ± 2.33cde	12.93 ± 1.95def	9.88 ± 1.77defg	51.16 ± 10.48ab	0.51 ± 0.19ab	419.31 ± 87.55b
TVSU-633	72.14 ± 26.11bc	25.54 ± 10.79de	21.02 ± 2.88cde	15.19 ± 1.64bc	14.03 ± 2.14ab	11.29 ± 2.26a	45.24 ± 13.61cde	0.42 ± 0.16 cd	307.82 ± 142.61cde
TVSU-1520	50.99 ± 22.12efg	24.03 ± 7.92e	20.30 ± 2.57def	14.10 ± 1.77de	12.52 ± 1.84 fg	9.12 ± 1.65 h	38.49 ± 12.24f.	0.25 ± 0.2f.	229.80 ± 95.32e
TVSU-939	60.71 ± 20.97de	32.53 ± 11.75b	18.82 ± 3.06 fg	14.07 ± 2.39de	13.45 ± 1.87bcde	10.01 ± 2.04bcdefg	52.78 ± 14.17a	0.46 ± 0.20bcd	291.74 ± 82.57cde
TVSU-513	57.21 ± 18.21ef	25.95 ± 8.26de	19.96 ± 3.55ef	14.33 ± 2.44cde	13.05 ± 1.17cdef	9.90 ± 1.28defg	44.14 ± 10.71de	0.31 ± 0.12ef	288.98 ± 81.75cde
TVSU-455	92.65 ± 30.96a	41.09 ± 12.66a	21.60 ± 3.01 cd	15.15 ± 2.19bc	13.75 ± 1.86abc	10.74 ± 2.28ab	52.48 ± 14.99a	0.57 ± 0.25a	550.26 ± 117.89a
TVSU-643	56.73 ± 22.02efg	23.68 ± 8.61e	20.87 ± 3.92cde	14.87 ± 2.32bcd	13.44 ± 1.75bcde	10.76 ± 1.68ab	45.43 ± 11.62cde	0.30 ± 0.11ef	252.46 ± 123.71de
TVSU-2096	71.12 ± 31.43bcd	27.95 ± 13.59bcde	23.07 ± 3.54a	15.39 ± 1.61ab	13.63 ± 1.38abcd	10.42 ± 1.11bcde	49.04 ± 11.42abcd	0.43 ± 0.22 cd	370.12 ± 174.23bc
TVSU-194	71.19 ± 28.85bc	26.77 ± 9.82de	22.47 ± 3.10ab	16.29 ± 2.16a	14.30 ± 2.12a	10.71 ± 1.88abc	41.91 ± 12.98ef	0.39 ± 0.20d	377.33 ± 140.91bc
TVSU-1611	49.88 ± 20.34 fg	25.03 ± 10.09de	20.86 ± 3.90cde	13.44 ± 2.22ef	12.92 ± 1.61def	9.98 ± 1.77cdefg	49.86 ± 13.77abc	0.30 ± 0.16ef	230.06 ± 107.18e
TVSU-1920	80.42 ± 21.98b	29.19 ± 8.40bcd	20.85 ± 2.00cde	15.55 ± 2.09b	14.02 ± 1.64ab	10.61 ± 1.25abcd	44.59 ± 9.41cde	0.51 ± 0.19abc	337.51 ± 150.78bcd
TVSU-1531	46.66 ± 23.13 g	27.58 ± 15.55cde	17.56 ± 2.82 g	12.87 ± 2.33	12.14 ± 1.64 g	9.32 ± 1.40gh	49.82 ± 14.25abc	0.31 ± 0.22ef	220.10 ± 87.20e
TVSU-1392	62.24 ± 19.55cde	26.16 ± 9.24de	20.31 ± 3.15def	14.64 ± 1.73bcd	12.70 ± 1.36efg	9.76 ± 1.25efgh	45.86 ± 9.29bcde	0.38 ± 0.13de	224.67 ± 128.70e
MIN	9.30	5.00	12.04	7.05	9.02	7.00	13.97	0.02	24.3
MAX	176.40	100.0	31.15	21.08	20.00	18.01	94.59	1.80	702.4
Grand means	64.99	28.12	20.94	14.59	13.24	10.16	47.15	0.39	310.6
CV	36.82	37.65	15.64	14.59	13.02	16.68	25.55	46.02	36.53
MS	8203***	859.0***	100.92***	31.446***	17.725***	15.110***	19,265***	0.348***	71,190***
LSD	36.912	17.354	4.091	2.765	2.064	1.945	13.936	0.280	248.95

*TNP* total number of pods, *FPS* final plant stand, *FSW* fresh seed weight (g), *NSP* number of seeds per pod, *YPP* yield per plant (g), *HSW* hundred seed weight (g), *YPL* yield per plot (g), *DSW* dry seed weight (g), *FPW* fresh pod weight (g), *MPN* mature pod number per plant, *LOP* length of pods(mm), *WOP* width of pods (mm), *LOS* length of seeds (mm), *WOS* with of seeds (mm), *SP* shelling percentage (%), *HI* harvest index, *YPU* yield per plot of unshelled. *MS* mean square, *LSD* least significant difference.

### Principal component analysis

**Figure 1:**
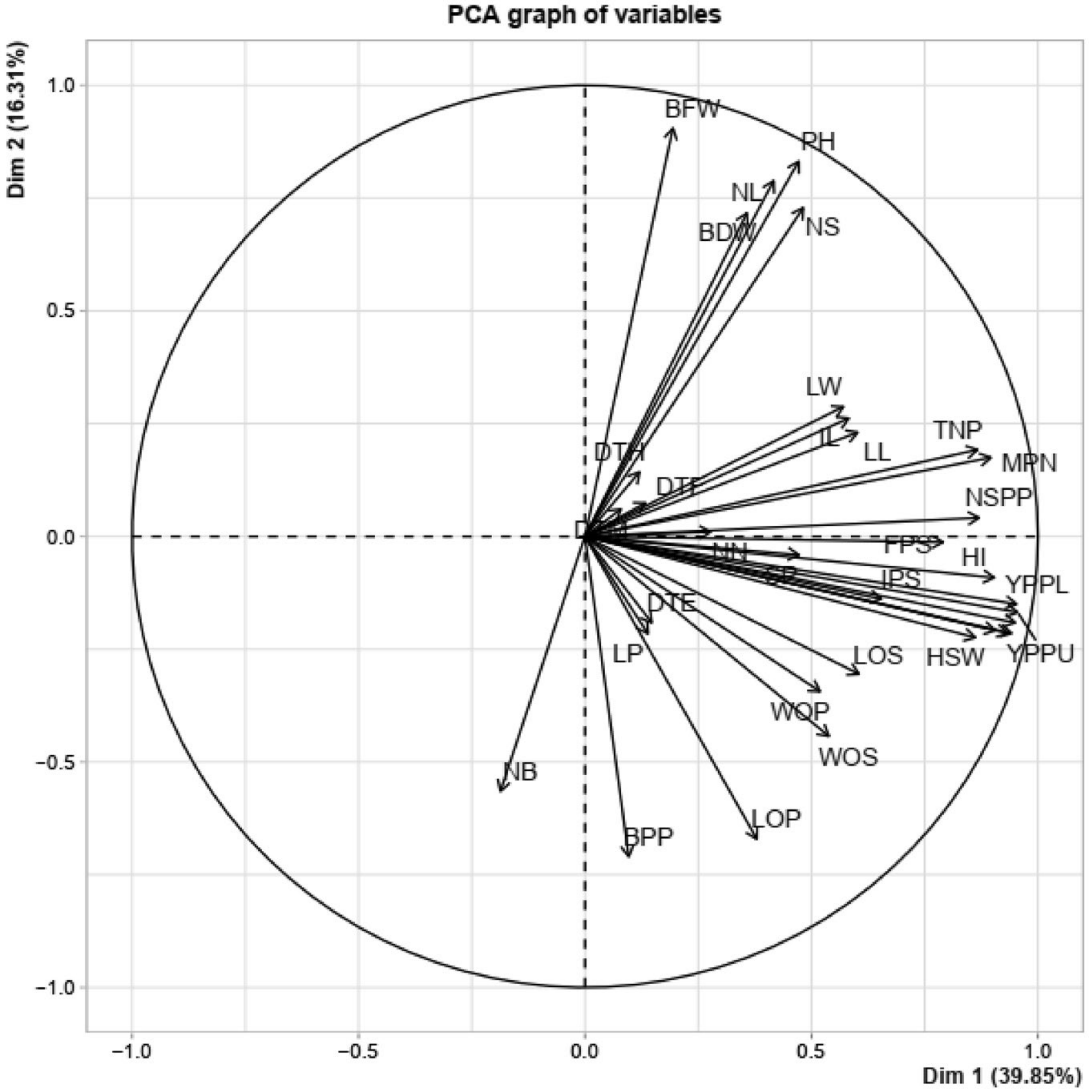
Principal Component Analysis (PCA) of quantitative trait scores. IL50%: internode length at 50% flowering, IPS50%: initial plant stand, LL50%: leaf length, LW50%: leaf width, PH50%: plant height, NL50%: number of leaves per plant, NS: number of stem per plant, LP50%: petiole length per stem, Min: minimum across the environment, Max: maximum across the environment, BFW: biomass fresh weight per plant, BDW: biomass dry weight per plant, BPP: biomass per plant (BDW/BFW X 100). TNP: total number of pods, FPS: final plant stand, FSW: fresh seed weight, NSPP: number of seeds per plant, YPP: yield per plant, HSW: hundred seed weight, YPPL: yield per plot, DSW: dry seed weight, FPW: fresh pod weight, MPN: mature pod number per plant, LOP: length of pods(mm), WOP: width of pods, LOS: length of seeds, WOS: with of seeds, SP: shelling percentage, HI: harvest index, YPPU: yield per plot of unshelled. DTE: days to emergence, DTF: days to flowering, DTM: days to maturity, DTH: days to harvest, NB: number of branches per plant, PL: panicle length per stem, NN: number of nodes per stem.

**Figure 2:**
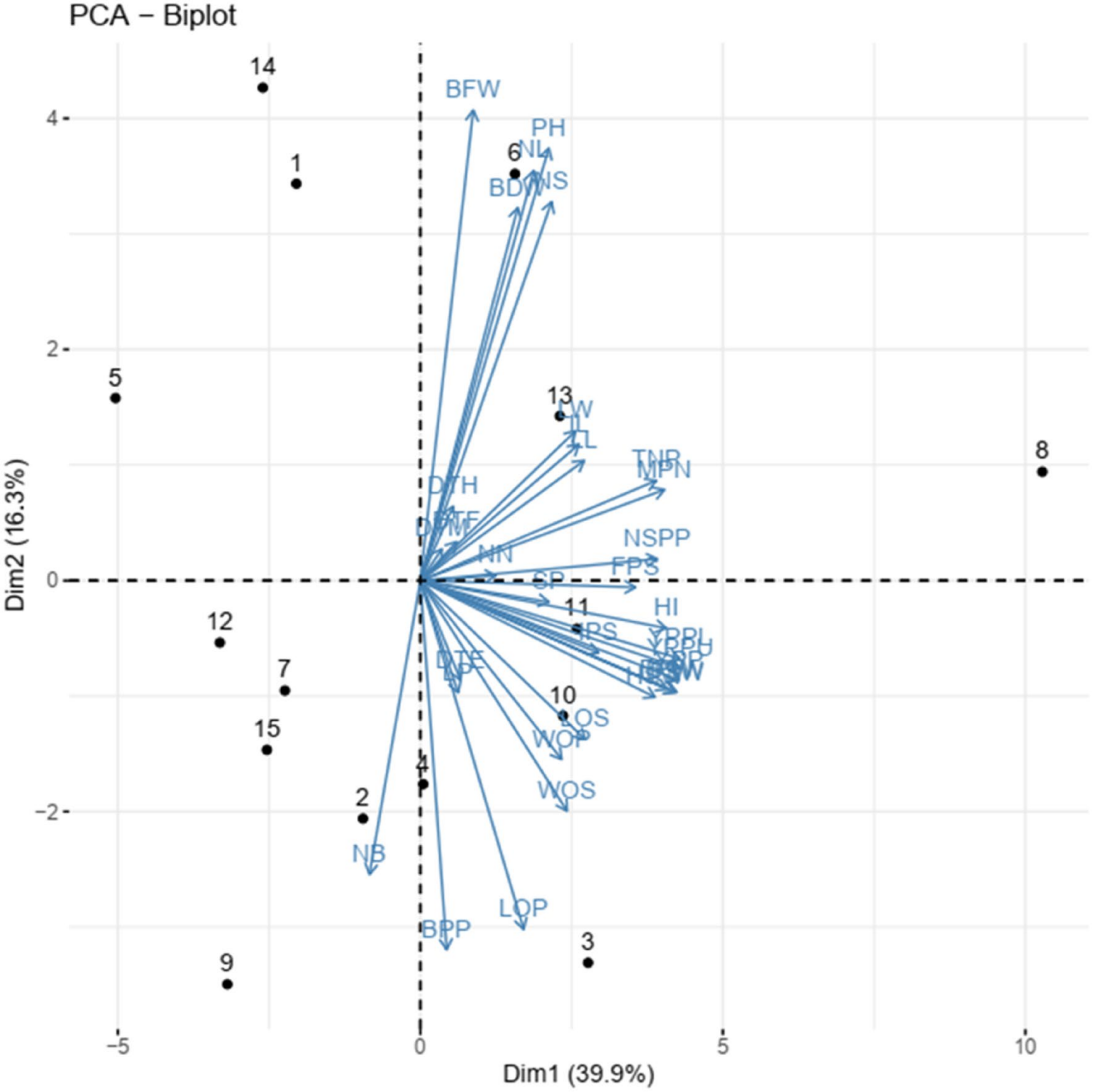
Dim 1 and Dim 2 biplot using morphological, phenological, and agronomic trait scores of the Bambara groundnut accessions. 1 = TVSU-454, 2 = TVSU-158, 3 = TVSU-438, 4 = TVSU-633, 5 = TVSU-1520, 6 = TVSU-939, 7 = TVSU-513, 8 = TVSU-455, 9 = TVSU-643, 10 = TVSU-2096, 11 = TVSU-194, 12 = TVSU-1611, 13 = TVSU-1920, 14 = TVSU-1531, 15 = TVSU-1392.

**Figure 3:**
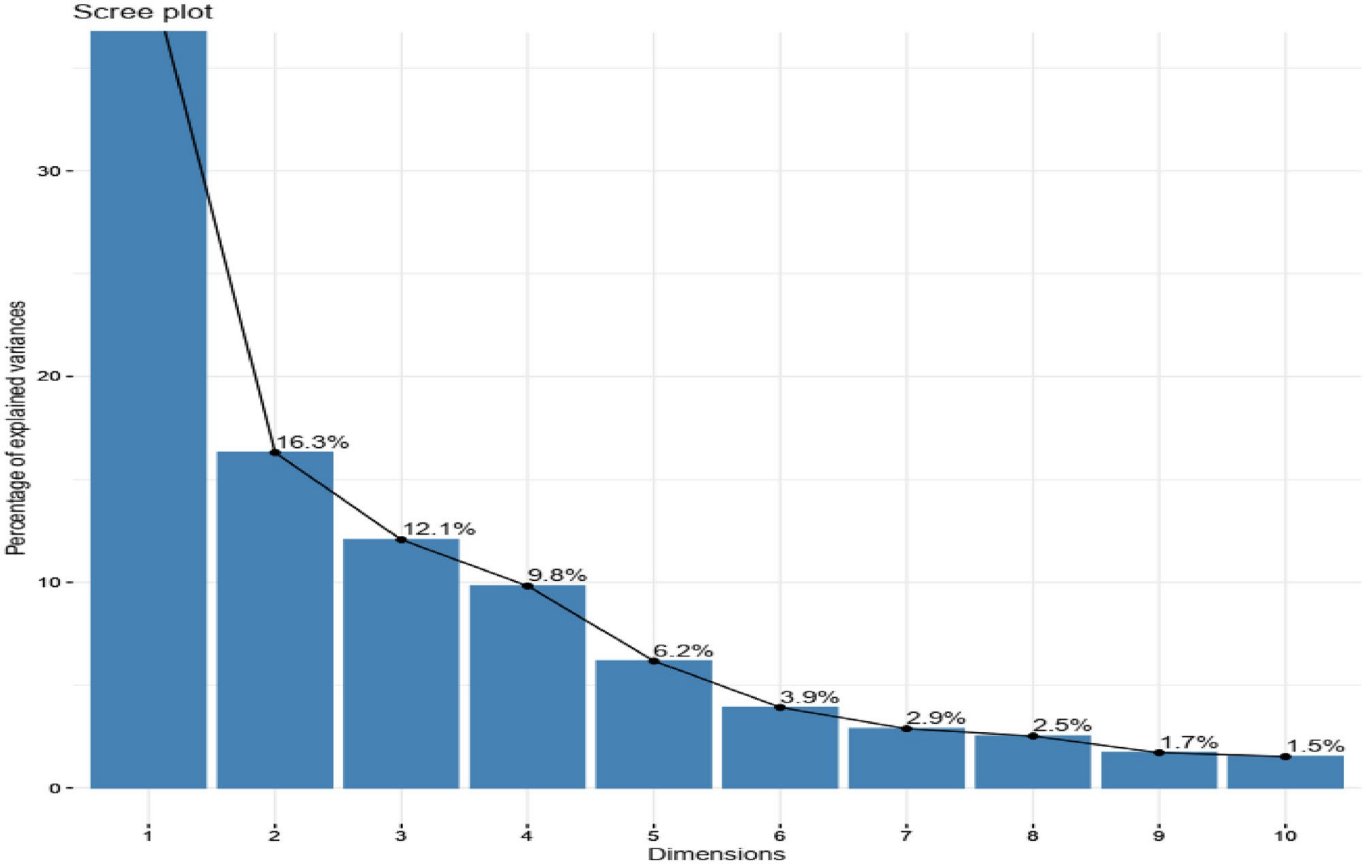
Percentage of explained variances of 10 PCs using quantitative trait scores of the 15 Bambara groundnut accessions.

**Figure 4:**
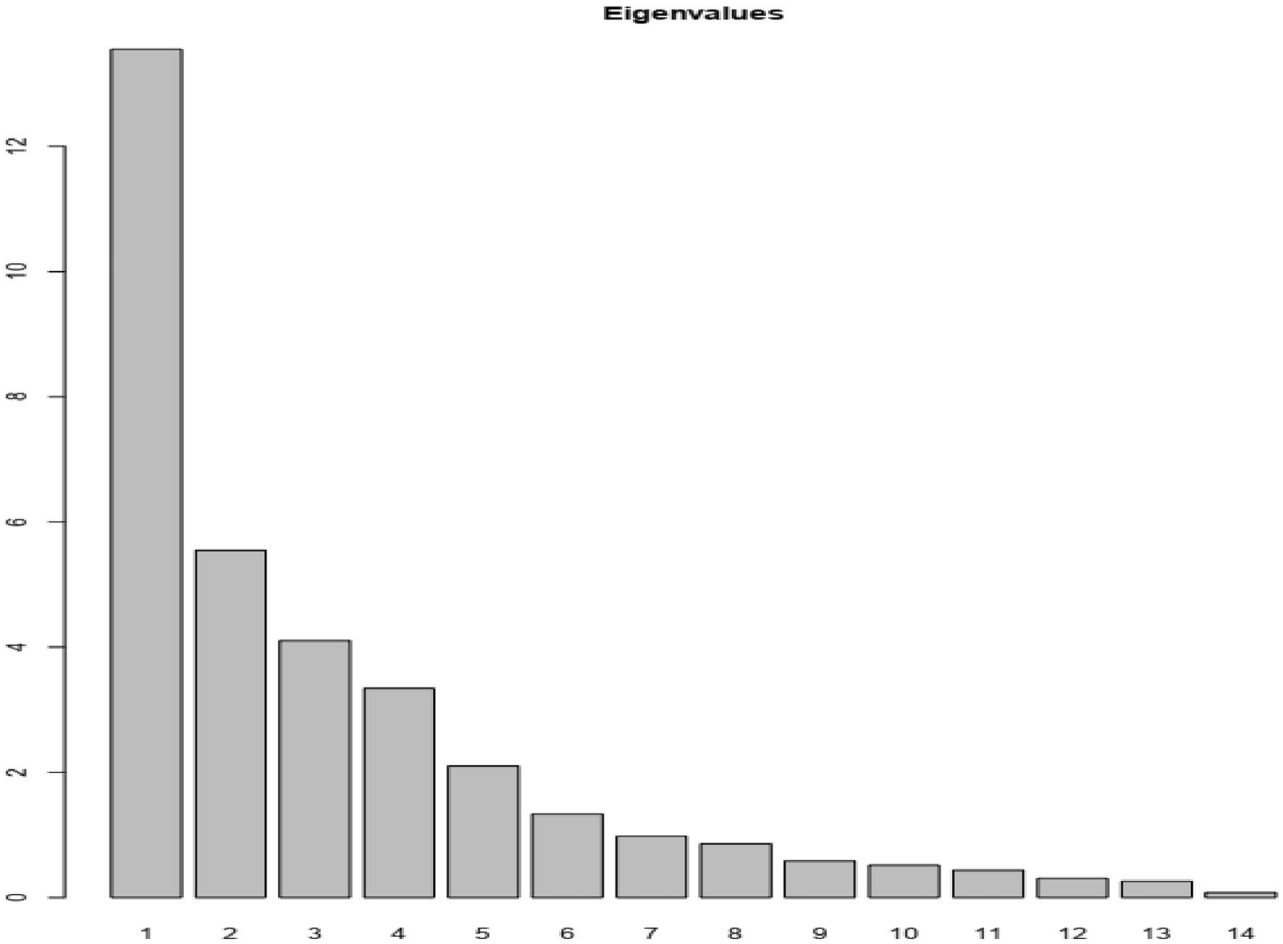
Eigenvalues of the quantitative parameters on the axes.

The Principal Component Analysis (PCA) with combined vegetative, phenological, and yield characters allows us to measure the relationships between variables and thus identified 10 dimensions (PCA), which significantly explained up to 96.76% of the variance in the data resulting in a strong contribution to the total variation (Figs. 1 and 2). The first two dimensions explained 56.16% of the variation (Figs. 1 and 3) with each dimension accounting for 39.85% and 16. 31% variation, respectively. Total number of pods, final plant stand, fresh seed weight, number of seeds per pod, yield per plant, hundred seed weight, yield per plot, dry seed weight, fresh pod weight, mature pod number per plant, width of pods, length of seeds, width of seeds, harvest index, yield per plot, internode length, initial plant stand, leaf length and leaf width had a positive correlation with Dim1 while biomass fresh weight per plant, plant height, number of leaves per plant, number of stem per plant, and biomass dry weight per plant had a positive correlation with Dim2 (Supplementary Table S1, Table 5, Figs. 1 and 3). Also, the following traits (days to emergence, days to flowering, days to maturity, and days to harvest), (width of pods, length of seeds, and width of seeds) and (leaf length and lumber of nodes per stem) had a positive correlation with PC3, PC4, and PC5, respectively (Supplementary Table S1, Table 5, Figs. 1 and 3). Among the variables, total number of pods, final plant stand, fresh seed weight, number of seeds per pod, yield per plant, hundred seed weight, yield per plot, dry seed weight, fresh pod weight, mature pod number per plant, width of pods, length of seeds, width of seeds, harvest index, yield per plot, internode length, initial plant stand, leaf length and leaf width were the major contributing characters in Dim1, for PC2 (biomass fresh weight per plant, plant height, number of leaves per plant, number of stem per plant, and biomass dry weight per plant), for PC3 (days to emergence, days to flowering, days to maturity, and days to harvest), for PC4 (width of pods, length of seeds, and width of seeds) and PC5 (leaf length and number of nodes per stem), highly contributed to the respective PCs (Table 5, Figs. 1 and 3). The rapport among dimensions and their proportion of variation and eigenvalues are presented in Figs. 2 and 4 and Table 5. For the first five principal components, the eigenvalues range from 13.5 for PC1 with the highest value to 2 for PC5, which recorded the lowest (Fig. 4 and Table 5).

**Table 5:** Eigenvalues, Proportion of variance (%), Cumulative variance (%), and trait contributions for the first six principal component axes for 34 phenotypic traits of Bambara groundnut accessions.

	Dim.1	Dim.2	Dim.3	Dim.4	Dim.5
Eigenvalue	13.50	5.50	4	3.50	2
Proportion of variance (%)	39.9	16.3	12.1	9.8	6.2
Cumulative variance (%)	39.9	56.2	68.3	78.1	84.3
IL	2.5076	1.2372	0.1822	7.1055	2.5453
LL	2.6761	0.9580	0.4381	0.2082	15.4599
LW	2.4033	1.4910	0.3746	1.9520	0.6538
PH	1.6441	12.4625	0.6886	0.5301	0.0931
IPS	3.1615	0.3478	0.7500	6.0014	9.6942
BFW	0.2786	14.7567	0.1627	0.3180	0.0049
BDW	0.9441	9.2618	2.3255	0.0042	3.1493
BPP	0.0690	9.0905	2.5155	2.7758	6.0731
LP	0.1424	0.8390	6.9714	5.3888	4.9235
NL	1.2802	11.2003	0.0145	1.2993	0.0738
NS	1.7153	9.5658	0.1843	1.6882	0.6394
NB	0.2572	5.7601	5.2549	2.2309	6.8996
NN	0.5685	0.0017	0.5817	9.0235	12.9638
TNP	5.5460	0.6649	0.0117	3.1969	3.1233
FPS	4.6115	0.0030	0.2067	3.7825	1.3241
FSW	6.5489	0.8320	0.1721	0.0002	1.0494
NSP	5.5910	0.0304	0.1426	3.9456	4.1998
YPP	6.6631	0.6604	0.1050	0.0430	0.9720
HSW	5.4847	0.9075	0.7216	1.4552	0.0066
YPL	6.7077	0.4069	0.0558	1.0582	0.0154
DSW	6.4406	0.8305	0.2232	0.0194	1.3834
FPW	6.0640	0.7858	0.0754	1.3917	0.3516
MPN	5.9335	0.5492	0.0189	2.6935	2.3925
LOP	1.0635	8.1168	0.0212	0.4537	2.3541
WOP	1.9888	2.1320	0.9502	12.0161	5.3763
LOS	2.7015	1.6810	0.0329	11.9623	1.1329
WOS	2.1424	3.5400	0.6266	9.6921	0.7355
SP	1.6487	0.0307	6.7837	7.4972	3.6574
HI	6.0303	0.1512	1.0844	0.1401	3.1926
YPU	6.7387	0.5115	0.3659	0.1110	0.0345
DTE	0.1610	0.6616	12.2666	0.1166	2.7002
DTF	0.1322	0.0991	14.8025	0.0127	0.2793
DTM	0.0469	0.0659	21.0259	1.2598	1.1323
DTH	0.1073	0.3673	19.8633	0.6264	1.4131

In PCA biplot (Fig. 3), both individual variables and genotypes are loaded at the same time indicating the relationship among traits and the distances between genotypes. The closer the vectors, the stronger the correlation. The PC 1 includes individual varieties accessions TVSU-455, TVSU-438, TVSU-2096, TVSU-194 and TVSU-1920. TVSU-455 was opposed to TVSU-1520.

### Correlation analysis of the traits

**Figure 5:**
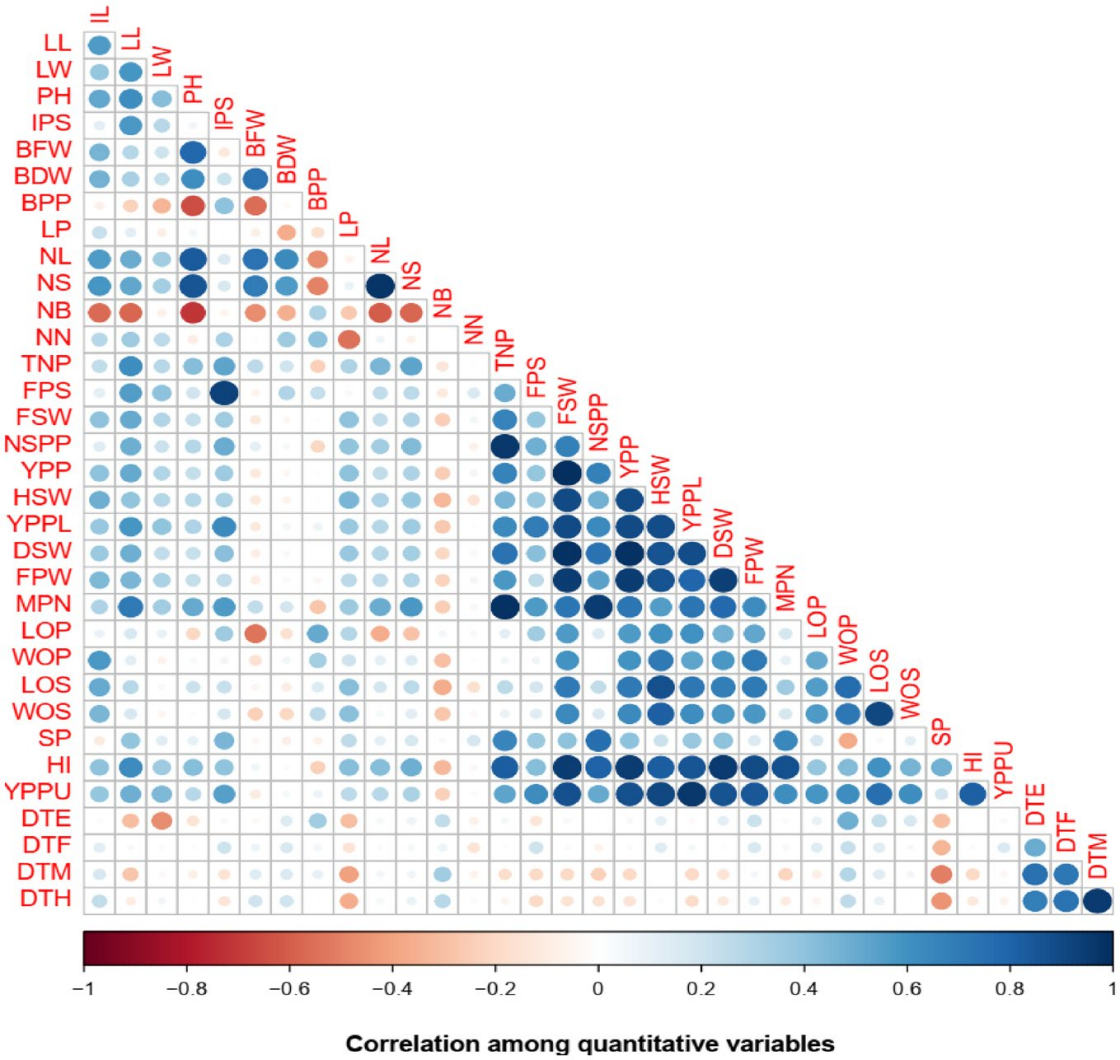
Correlations among the phenological, morphological, and agronomic traits; Pearson’s rank correlation matrix indicating the correlation among variables of Bambara accessions grown across three environments.

The correlation coefficients for 34 traits including growth, phenological, and yield characters are presented in Fig. 5. There were significant differences among the correlations of the 34 traits. According to the scale in Fig. 5 the non-significant or significant differences (*p* < 0.05) are shown with blue or red rounds which represent negative and positive correlations, respectively. The more intense the colors of the relationship, the stronger the correlations. Among the positive correlations, however, correlations with leaf length, leaf width, petiole length, and chlorophyll content were not significant (Supplementary Tables S2, S3). Leaf length had strong and positive correlation with leaf width (r = 0.58, *p* = 0.02*), plant height (0.61*), initial plant stand (r = 0.57*), number of stem per plant (r = 0.51*), total number of pods (r = 0.62*), final plant stand (r = 0.56*), fresh seed weight (r = 0.51*), yield per plant (r = 0.51, p = 0.05*), yield per plot (r = 0.58*), MPN (r = 0.7**), and harvest index (r = 0.63*). Plant height had positive correlation with internode length (r = 0.51*), leaf length (r = 0.61*), leaf width (r = 0.42, *p* = 0.12), biomass fresh weight per plant (r = 0.79****), biomass dry weight per plant (r = 0.61, *p *= 0.02*), number of leaves per stem (r = 0.84***), number of stem per plant (r = 0.86***), total number of pods (r = 0.425), final plant stand (r = 0.20), fresh seed weight (r = 0.24), number of seeds per pod (r = 0.29, *p* = 0.30), yield per plant (r = 0.24), hundred seed weight (r = 0.29), yield per plot (r = 0.31), dry seed weight (r = 0.23), fresh pod weight (r = 0.23), mature pod number per plant (r = 0.5), length of seeds (r = 0.21), harvest index (r = 0.42), and yield per plot of unshelled (r = 0.27). Total number of pods had a strong, positive, and highly significant correlation with final plant stand (r = 0.49), fresh seed weight (r = 0.67***), number of seeds per pod (r = 0.96*****), yield per plant (r = 0.67***), hundred seed weight (r = 0.46), yield per plot (r = 0.64**), dry seed weight (r = 0.74***), fresh pod weight (r = 0.58*), mature pod number per plant (r = 0.98*****), shelling percentage (r = 0.66, ***), harvest index (r = 0.83****), and yield per plot of unshelled (r = 0.52*). A perfect positive significant correlation (r = 1.00) was observed between yield per plant and hundred seed weight, meanwhile a positive and moderate and equal correlation was recorded with the characters of harvest index and leaf width (r = 0.36); fresh seed weight and initial plant stand (r = 0.35), yield per plant and Initial plant stand (r = 0.35), Mature pod number per plant and Length of seeds (r = 0.35). Fresh seed weight had a very strong, positive, and highly significant correlation with number of seeds per pod (r = 0.68*), yield per plant (r = 1, *p* = 0.000), hundred seed weight (r = 0.89****), yield per plot (r = 0.98****), dry seed weight (r = 0.94****), fresh pod weight (r = 0.94****), mature pod number per plant (r = 0.72***). Yield per plant had a very strong, positive, and very highly significant correlation with hundred seed weight (r = 0.89****), yield per plot (r = 0.98****), dry seed weight (r = 0.94****), fresh pod weight (r = 0.94****), mature pod number per plant (r = 0.72***), harvest index (r = 0.85***), yield per plot of unshelled (r = 0.90****) (Supplementary Tables S1, S2).

### Clustering analysis

**Figure 6:**
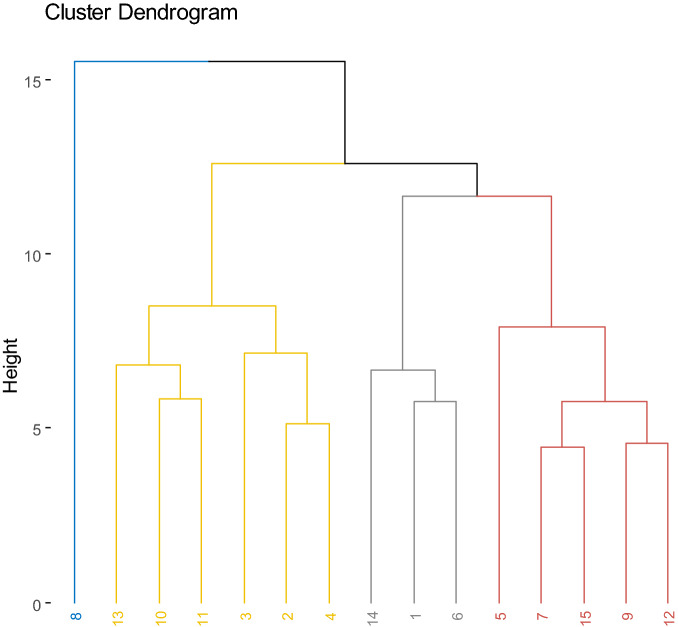
Hierarchical clustering dendrogram of the 15 bambara groundnut accessions based on quantitative traits. 1 = TVSU-454, 2 = TVSU-158, 3 = TVSU-438, 4 = TVSU-633, 5 = TVSU-1520, 6 = TVSU-939, 7 = TVSU-513, 8 = TVSU-455, 9 = TVSU-643, 10 = TVSU-2096, 11 = TVSU-194, 12 = TVSU-1611, 13 = TVSU-1920, 14 = TVSU-1531, 15 = TVSU-1392.

The clustering analysis produced a dendrogram categorizing the 15 accessions into 4 groups based on the morphological, phenological, and agronomic traits (Fig. 6). Cluster I consisted of one accession (TVSU-455). Cluster II comprised six accessions including TVSU-2096, TVSU-194, TVSU-1920, TVSU-158, TVSU-438, and TVSU-633. Cluster III is made up of three accessions, namely TVSU-1531, TVSU-454, and TVSU-939. Cluster IV included four accessions including TVSU-1520, TVSU-513, TVSU-643, TVSU-1392, and TVSU-1611.

Cluster I illustrated by only one accession TVSU-455 was distinguished by the highest mean values for the total number of pods, final plant stand, fresh seed weight, number of seeds per pod, yield per plant, hundred seed weight, yield per plot, dry seed weight, fresh pod weight, mature pod number per plant, the width of pods, width of seeds, shelling percentage, harvest index, yield per plot of the unshelled, initial plant stand, number of leaves per plant, number of stem per plant, biomass dry weight per plant, and biomass per plant; recorded across the three experimental locations. On the other hand, cluster II was characterized by the highest mean values for Length of pods, Length of seeds, Width of pods, Internode Length, Leaf length, Leaf width, Petiole length per stem and biomass per plant apart from these characteristics were next best to cluster I in terms of yields and yield components. However, cluster IV was characterized by low mean values of yield per plant, hundred seed weight, yield per plot, dry seed weight, mature pod number per plant, length of seeds, with of seeds, and yield per plot of unshelled. This cluster IV was majorly made up of accessions from unknown origins while the best accession TVSU-455 performing across the three environments is from Cameroon.

### Qualitative trait analysis

**Figure 7:**
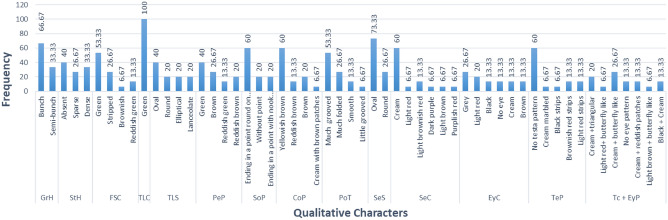
Frequency dispersal of the measured qualitative characters of Bambara groundnut varieties. Growth Habit GrH; Stem Hairiness StH; First Stem Color FSC; Terminal leaflet color TLC; Terminal leaflet shape TLS; Petiole pigmentation PetP; Shape of pods SoP; Color of pods CoP; Pod texture PoT; Seed shape SeS; Seed color SeC; Eyes color EyC; Testa pattern TeP; Testa color + eye pattern TC + EyP.

**Figure 8:**
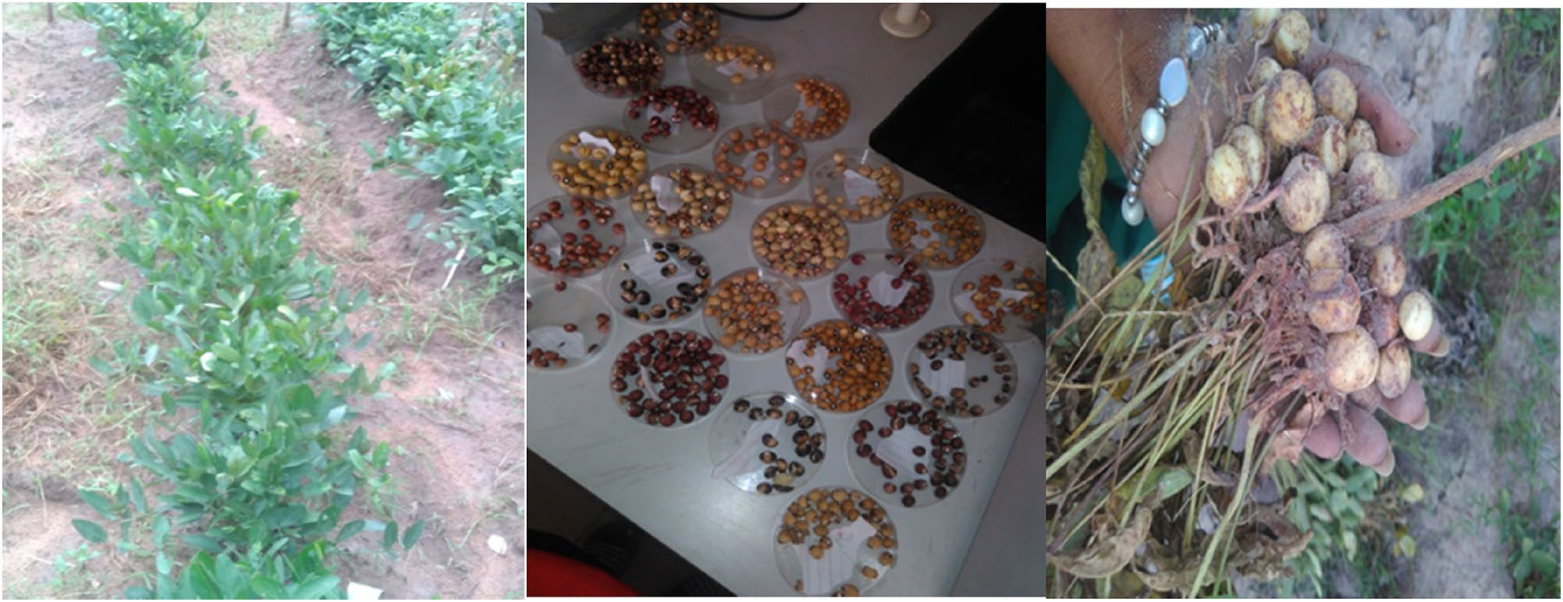
Qualitative trait variation.

Out of the 15 accessions used during this research, 66.67% had a bunch type of growth habit while the remaining 33.33% had a semi-bunch type of growth habit (Fig. 7). It was also observed that 40% had hair on their stems, 33.33% had a large amount of hair on the stem while the remaining 26.67% didn't have hair on their stems (Fig. 8). In addition, most of the accessions had a green first stem color of 53.33% followed by stripped 26.67%, then reddish green 13.33% while little accessions had a brownish 6.67 first stem color. All the accessions exhibited 100% terminal leaflet color of Green. The terminal leaflet shape was oval, round, elliptical, and lanceolate with frequencies of 40%, 20%, 20%, and 20%, respectively. Most accessions had a green petiole color of 40%, followed by 26.67% for brown petiole, then 20% for reddish brown petiole, and few had a reddish green petiole with a frequency of 13.33. 60% had pods that ended in a point and round on the other side, 20% were without a point while the last 20% had point but ended with a nook on the other side. The color of the pod varied from yellowish brown 60%, reddish brown 13.33%, brown 20% and cream with brown patches 6.67%. The accessions had different textures of pods after they were harvested. 53.33% had a much-grooved pod, 26.67% had a much-folded pod, 13.33% had a smooth pod, and a few percent, 6.67% had a little grooved pod. In this regard also, they exhibited two different shapes of seeds which were oval and round with frequencies of 73.11% and 26.67%, respectively. The seed color and their frequency ranged from cream 60%, light red 6.67%, light brownish-red 13.33%, dark purple 6.67%, light brown 6.67%, and to purplish red 6.67%. Some accessions 13.33% did not have eye color, 26.67% had grey, 20% had light red, 13.33% had black eye color, the next 13.33% had cream and the last 13.33% had a brown eye color. Similarly, 60% of the accessions didn’t have a test pattern, 6.67% had a cream marbled pattern, 6.67% had black stripes, 13.33% had brownish red strips and the last 13.33% had light red stripes. Observations were also noted on their testa color and eye pattern around the hilum. 20% had cream and triangular, 6.67% had light red and butterfly-like, 26.67% had cream and butterfly-like, 13.33% had no eye pattern, 13.33% had cream and reddish patches, 6.67% had light brown and butterfly-like pattern and the remaining 13.33% had a black and cream testa (Supplementary Fig. S1).

## Discussion

### Soil characteristics

There were different types of soils in terms of texture, and physical and chemical properties from the three environments (Bowen, Ibadan, and Odeda) under study. It is ascertained that the climatic and soil conditions influence the growth, development, and yield of crops. Crops also respond differently to different types of soils. It has been observed that Bambara groundnut has a high yield in sandy soils because it bears fruits underground and sandy soil has porous and loose structures with large pores which allow for pods to grow and smooth harvest. When the sandy soil gets dried, they produce thin, loose fissures which are advantageous, especially in the semi-arid tropics where there’s an uneven rainfall pattern and long droughts. Clay soil has a high water retention ability and it expands when wet and vice versa when dry.

### Agro-morphological characteristics

With other required factors kept in place, the ability of a crop to compete with weeds, withstand drought and other harsh climatic and environmental conditions, yield improvement, and high nutrient uptake and utilization are some factors contributing to crop productivity^35^. This reports are similar to the findings of Khan et al.^72^. The significant difference that was observed among the accessions indicates that there is a high level of variation in the selected accessions. The variations in traits bring about the selection of best lines for improvement^36^. However, according to Aremu et al^39^, the most dominant source of variation is the environment and it is of high importance in plant breeding. There is a high level of variation among the germplasm of the plant itself and these must be evaluated to develop cultivars of those germplasms^40^. Also, there is a high level of variation even within the same accessions and across different accessions and these variations are further manifested in the environments in which they are grown^35^.

Bambara groundnut has been localized in various environments and its significance especially in the Sub-Saharan part of Africa is increasing because it is a crop that has a rich source of diversity. In this current study, all 15 accessions of Bambara groundnut showed a very high level of diversity and variability for all the parameters studied. Our findings are similar to those of^41–44^. In the publication of Khan et al.^2^, they all reported a coefficient of variation (CV %) ≥ 20% for traits like petiole length, number of pods, hundred seed weight, and yield. This report also confirms a coefficient of variation (CV %) ≥ 20% for the same traits of petiole length (38.46%), number of pods (36.32%) and yield (38.74%) except for hundred seed weight which has a CV % of (15.20) < 20%. The high coefficient of variation observed in some of the traits in traits in this study shows that there is a high level of heterogeneity across the studied environments. This high heterogeneity in Bambara groundnut was also reported by Goli et al.^45^, Khan et al.^2^ and Khan et al^28^. The variations in the phonological traits and morphological traits are a result of the differences in the genetic makeup of the accessions and planting seasons^46^. For instance, in this present study, days to flowering range from 34 to 53 days and Khan et al.^28^ reported 36–53 days which is still in the present range which are both comparably lower to those reported by Masindeni^47^ 43–80 days, Goli et al.^45^ 38–68 days and comparably higher than those reported by Quadraogo et äl.^48^, 32–42 days. A significant difference was reported by this present study in the number of days to maturity which ranges from 89 to 118 days and it agrees with the reports from Goli et al.^45^ and Masindeni et al.^47^.

For effective breeding for Bambara groundnut, there is a need to study the GEI for the crop in order for the breeders to identify the stable genotypes across the locations or the particular genotypes that will do better in particular environments^49^. There are various factors that affect the responses of genotypes to locations and planting seasons some of which include soil fertility, pests and diseases, rainfall, humidity, and temperature. This present study showed that the responses of growth traits especially the morphological traits are strongly affected by accessions and locations and this is by the research of^50^ who also experimented with three different locations. This very highly significant effect observed for traits like number of branches, number of nodes, leaf length, leaf width, number of stems, number of leaves and internode length can be attributed to differences in climatic and soil conditions exhibited at the three locations. This further buttresses the need for accessions to be evaluated under different environments to identify the most stable and the highest yielding varieties like TVSU-455 and this agrees with the reports of researchers like Rubilar et al.^51^, and Olanrewaju et al.^52^ Also, the accessions in this present research showed that there is no significant variation in plant height, which is absolutely in support of^1,53^. All the seventeen yields and yield-related traits evaluated in this study showed a very high significant genetic discrepancy. A similar report was given by Shegro et al.^53^, who stated that these variations were accredited to the effect of genotype by environment interaction on Bambara groundnut yield. The traits like total number of pods, fresh seed weight, dry seed weight, fresh pod weight, hundred seed weight, number of seeds per plant, and harvest index show very high significant differences and this was similarly reported by^53^. The hundred seed weight ranged from 76 to 125 g and this is a critical factor that is usually used to determine the morphological traits relating to plant yield^28,54^. The hundred seed weight also influences yield directly. In this study, the variations in seed length and seed width may be attributed to the different seed shapes, sizes, and shapes of the pods, while the variations in hundred seed weight can also be attributed to the size of the seeds and nutrient contents. The yield of Bambara groundnut was recorded from 146.6 to 2678.6 kg ha^−1^ by^43^, 1,058.8 kg ha^−1^ by^55^ and from 0 to 1266.77 kg ha^−1^ by^52^ whereas in this study we reported from 997.3 to 1106.4 kg ha^−1^ for the shelled yield and from 1912.9 to 2300.8 kg ha^−1^ for the unshelled yield. The findings from this study and other studies by previous researchers show that there is a high level of diversity and a high level of influence of the environment on the growth, development, maturity and yield of Bambara groundnut.

### Principal component analysis

The principle components allow identification of quantitative traits that are highly and strongly correlated with each component. Additionally, PC is for the classification of genetically similar accessions into the same groups playing a similar function as cluster analysis^56^. Additionally, Mercati et al.^57^; Figàs et al.^58^; Nankar et al.^59^ reported that cluster analysis is very useful in the classification of genotypes based on their similarity and affiliation. Valombola et al.^60^ demonstrated that resemblances of accessions could be because they might be the same accessions but have different names given by different ethnicities or cultivated from agro ecological zones. The breeding material should be selected from different clusters for the reason that each cluster has its specificity and this could help optimize the betterment of the newly developed varieties and hybrids in terms of performance.

Moreover, many studies^52,61^ have used PCA clustering analysis and multidimensional scaling to evaluate genetic variability and genetic diversity in crop accessions including Bambara. As in the previous studies^28,52^, we also found that Dim 1 accounted for the highest percentage of variance which was followed by the PC2 and this pattern was observed in descending order in the remaining 8 dimensions. Khan et al.^28^ reported a total variation at 45.88% for PC1 and 10.68% for PC2 while Olanrewaju et al.^52^ found 24.67% for PC1 and 17.63% for PC2, the two authors worked on Bambara. In this study, the PC1 accounted for 39.85% and the PC2 represented 16. 31% of the total variation.

### Correlation analysis

To select a genotype, it is of paramount importance to go through screening of genotypes and identify of the traits that are strongly and positively correlated. Karikari and Tabore^62^ reported that the understanding of variation and inter-correlation between traits is fundamental for fruitful selection. Similarly, Adebisi et al.^63^ believe that one should take into consideration the strong correlation of variables in the selection process of superior genotypes for crop improvement. The correlation coefficients for 34 traits including vegetative, phenological, and yield traits were assessed in this study. The R software packages provide R-values and the level of probability for their significance. Plant height had a positive correlation with internode length, leaf length, leaf width, biomass fresh weight per plant, biomass dry weight per plant, number of leaves per stem, number of stem per plant, the total number of pods, final plant stand, fresh seed weight, number of seeds per pod, yield per plant, hundred seed weight, yield per plot, dry seed weight, fresh pod weight, mature pod number per plant, length of seeds, harvest index, and yield per plot of unshelled. This indicates that plant height is interrelated with vegetative and reproductive traits. Similar results were reported by many authors including Khan et al.^28^, Olanrewaju et al.^52^. Total number of pods had a strong, positive, and highly significant correlation with final plant stand, fresh seed weight, number of seeds per pod, yield per plant, hundred seed weight, yield per plot, dry seed weight, Fresh pod weight, mature pod number per plant, shelling percentage, harvest index, and yield per plot of unshelled. Similar observations were made by Khan et al.^2^.

In this study, we observed strong and positive and high contribution between hundred seed weight and yield (for yield per plant and yield per plot 0.89 and yield per plot of unshelled 0.90), which is contrary to the results of Khan et al.^28^ but similar to those of Karikari and Tabore^62^, Misangu et al.^64^. The correlation matrix in this study also showed that yield was strongly and positively correlated with seed width, seed length, pod width, pod length, harvest index, shelling percentage, mature pod number, fresh pod weight, dry seed weight, number of seed per plant, fresh seed weight, fresh pod weight, total number of pods, leaf length, internode length, plant height, petiole length, number of the leaf. The correlation between yield and leaf length and the number of leaves demonstrated the ability of the plant to efficiently intercept light for photosynthesis but the yield was negatively correlated with biomass fresh, biomass dry weight, and biomass per plant, which could be because plant during seed development prioritized seed filling to the other plant organs. Similar results were reported by Evans^65^, Carter^66^, Helms^67^ who revealed that yield and photosynthesis are often poorly correlated, both in field crops and in forest trees., while the results of Khan et al.^1^ are contradictory because they reported that biomass fresh weight and biomass dry weight were correlated with yield. Though, in their last findings, Khan et al.^73^ demonstrated that there was significant correlation between several growth parameters, yields and yield components. On the other hand, in our study seed width, seed length, pod width, pod length, harvest index, shelling percentage, mature pod number, fresh pod weight, dry seed weight, number of seeds per plant, fresh seed weight, fresh pod weight, the total number of pods greatly contributed to the yield. Similar results were obtained by Karikari and Tabore^62^ who reported that the number of pods, number of seeds, and seed weight per plant had a strong influence on final seed yield while the results of^52^ were contradictory because they found that the numbers of pods, number of seeds, and total seed weight were positively correlated but negatively correlated with the yield. The seed size contribution to high yield cannot be overemphasized as farmers and consumers always seek big seeds and fruits, Duncan et al.^68^, Pathirana^69^, and Karikari and Tabore^62^ demonstrated that the size of the seed is well considered in the market either locally and internationally as an essential factor worldwide.

## Conclusion

This study allowed us to understand the effects of genotypes by environment interactions on the 15 Bambara groundnut accessions used. Moreover, the phenotypic expressions of the accession are the results of genotypic expression under the influence of the environment and there were significant responses of the 15 accessions to different locations of the experiments. Based on its high vegetative and yield performance across 3 locations, TVSU-455 is recognized as the best accessions for higher yields and can greatly contribute to food security in Nigeria. It can also be used in breeding programs to improve the accessions with low yields.

## Materials and methods

### Experiment location

The research was conducted from August 2021 to December 2021 at three different environments. The experiments were conducted at Bowen University teaching and research farm Iwo, Osun State, Nigeria (7°38′N, 4°11′E) with an altitude of 322 m above the sea level, a leased farmland in Ologuneru Ibadan, Oyo State, Nigeria (7°44′ N, 3°83′E) with an altitude of 275 m above sea level and a leased farmland in Odeda, Ogun state, Nigeria (7°23′N, 3°53′E) with an altitude of 162 m above the sea level. The seeds of the accession were sown in an open field across all environments during the 2021 cropping season. Presented in Table 6 are the temperature, humidity and rainfall for each of the experimental site.

**Table 6:** Average climatic conditions the three locations during the experiments.

Locations	Parameters	AUG	SEPT	OCT	NOV	DEC
OSUN (BOWEN)	Min temperature (°C)	23.3	23.4	17	23.6	25.6
	Average temperature	25.2	25.7	25.9	27.7	27.7
	Max temperature (°C)	26.9	27.5	27.7	29.9	29.5
	Average humidity (%)	80	81	86	76	65
	Average rainfall (mm)	200.4	224.6	164.1	44.4	11.7
OYO (IBADAN)	Min temperature (°C)	22.8	22.2	22.2	24.8	27.5
	Average temperature	25.8	26.4	27.0	28.6	29.5
	Max temperature (°C)	28.4	28.0	29.2	30.7	30.9
	Average humidity (%)	89	86	86	81	62
	Average rainfall (mm)	226.5	235.4	169.3	39.8	7.3
OGUN (ODEDA)	Min temperature (°C)	24.3	23.9	24.4	23.3	25.6
	Average temperature	25.7	26.1	26.9	27.5	28.1
	Max temperature (°C)	27.0	27.2	28.3	29.4	30.0
	Average humidity (%)	89	88	87	86	74
	Average rainfall (mm)	242.1	254.2	139.2	32.9	4.45

### Soil sampling and analysis

Top soil was collected from the field for sampling at a depth of 0–15 cm randomly over the entire plot in the three environments. Cutlass and hand trowel was used to dig and collect soil samples. The collected samples were put together and sorted per location in order to obtain a composite sample after the experiment was carried out. The collected soil sample was then taken to the university laboratory for analysis. The samples were dried under shade and ground in a glass mortar and pestle to ensure uniformity in nutrient distribution and for samples to be a true representation of the plots. After this process, the sample was sieved and the procedures for the chemical analysis and particle size distribution were carried out (sand, clay, silt, pH, organic carbon (OC), total N, exchangeable Ca, Mg, K, available P, Na, Mn, Cu, Fe, and Zn).

### Plant materials

Fifteen (15) accessions of Bambara groundnut were selected for this research work out of the Bambara groundnut germplasm that is located at the Genetic Resources Center, IITA, Ibadan Nigeria. The list of Bambara groundnut accessions used in this research is presented in Table 7. Five plants in the middle were selected to ensure uniformity across all beds and these five plants were used for data collection. The 5 plants were selected for data collection to avoid edge and border effects.

**Table 7:** The accessions of Bambara groundnut and their countries of origin.

S/N	Accessions	Origin
1	TVSU-454	Cameroon
2	TVSU-158	Ghana
3	TVSU-438	Cameroon
4	TVSU-633	Nigeria
5	TVSU-1520	Unknown
6	TVSU-939	Zambia
7	TVSU-513	Cameroon
8	TVSU-455	Cameroon
9	TVSU-643	Nigeria
10	TVSU-2096	Unknown
11	TVSU-194	Benin
12	TVSU-1611	Unknown
13	TVSU-1920	Cameroon
14	TVSU-1531	Unknown
15	TVSU-1392	Unknown

### Experimental design and intercultural practice

The experiment was conducted by using a randomized complete block design (RCBD) with three replications across all locations. RCBD was used because of fertility gradients of the experimental sites. In each replication, there were 15 plots/beds with each bed measuring 3 m × 0.5 m. The furrow spacing between each bed was 30 cm and the intra-spacing distance between plants was 30 cm while the interspacing distance between plants was 50 cm. The replications were separated from each other by a distance of 1 m. The total size of the experiment plot was 13 m × 12 m leaving 1 m of spacing before the first replication and 1 m spacing after the third replication and with 15 beds per replication and a total of 45 beds across all locations. Each replication had 11 plants per plot.

### Measurement of parameters for data analysis

For this study, the phonological, growth, yield traits, and qualitative data were taken (Tables 8 and 9).

**Table 8:** The list of 34 phenological, vegetative, and yield traits considered according to IPGRI, IITA, and BAMNET^71^. (Nos 1–4 are the phonological traits, Nos 5–17 are the vegetative traits while Nos 18–37 are the yield traits.

S/N	Name of traits	Code	Description and measurement type
1	Days to emergence	DTE	The no of days from planting to the arrival of the first typical leaf on the soil surface
2	Days to 50% flowering	D50%F	Measured from seed germination to arrival of 50% flowerings
3	Days to 50% maturity	D50M	The number of days from sowing to 50% maturity
4	Days to harvest	DTH	The number of days from sowing to the period of harvest
5	Plant height	PH	Taken at 30 days and 50%F. Measured from soil level to the tip of terminal leaflet
6	No of branches per plant	NBP	Data was counted at the time of harvest from the stems of the 5 measuring middle plants
7	No of stems per plant	NSP	Taken at 30 days and 50%F. Data counted from five middle healthy plants
8	Length of petioles per stem	LPS	Taken at 30 days and 50%F. Data counted from five middle healthy plants
9	No of leaves per plant	NLP	Taken at 30 days and 50%F. Data counted from five middle healthy plants
10	No of nodes per plant	NNP	Data counted at the time of harvest from the stems of middle healthy plants
11	Leaf length	LL	Taken at 30 days and 50%F. Measured from the longest leaf on the middle stem
12	Leaf width	LW	Taken at 30 days and 50%F. Measured from the widest leaf on the middle stem
13	Inter node ode length	IL	Taken at 30 days and 50%F. Average internode length of 3 middle stems of 5 plants
14	Panicle length	PL	Data measured at 50%F from the length of 2 flowers on stems of 5 healthy middle plants
15	Biomass fresh weight	BFW	Weight recorded at harvest of five healthy plants that were used in measuring
16	Biomass dry weight	BDW	Weight of dried plants in the oven at 35 °C for 2 h from the same harvested plants
17	Biomass per plant	BPP	Obtained by dividing dry by wet biomass and multiplying by 100 for the same plants used
18	Total no of pods per plant	TNP	Data was counted at harvest from the five middle plants that were used during morphology
19	Mature pod no per plant	MPN	Data was counted after harvest and was recorded and separated from immature pods
20	Immature pod no per plant	IMP	Data was counted after harvest and separated from five healthy middle plants
21	Fresh pod weight	FPW	Recorded at the time of harvest by using an Atom A-120 standard measuring scale
22	Fresh seed weight	FSW	Recorded after breaking the pods and weighed using the same measuring scale
23	Dry seed weight	DSW	Weighed and recorded after seeds have been oven dried and set at 12% moisture
24	Length of pods	LOP	Measured within 3 days of harvesting by using a manual caliper. Pods were set horizontally
25	Width of pods	WOP	Measured within 3 days of harvesting by using a manual caliper. pods were set vertically
26	Length of seeds	LOS	Measured for 5 plants by using a manual caliper and the seeds were placed horizontally
27	Width of seeds	WOS	Seeds were placed vertically and were measured by using a manually operating caliper
28	Shelling percentage	SP	Measured by dividing the fresh seed weight with the fresh pod weight multiplied by100
29	Number of seeds per pod	NSP	Counted and recorded at harvest after breaking of pods
30	Yield per plant	YPP	Weighed after breaking the pods per plant by using a weighing scale
31	Hundred seed weight	HSW	Data weighing of 100 fresh seeds by using a weighing scale at harvest
32	Yield per plot (Shelled)	YPPs	Yield of all the harvested plants on each plot after breaking the pods
33	Yield per plot (Unshelled)	YPU	Yield of all the harvested plants on each plot before breaking the pods
34	Harvest index	HI	Measured using the formula of ratio of grain yield and biological yield
35	Yield kg per hectare	Yield(Kg/ha)	The dry seed weight of all the harvested plants (Yields) across all blocks and replications and then converting into kg/ha

**Table 9:** The qualitative traits measured according to IPGRI, IITA, and BAMNET^71^, and Khan et al.^28^.

S/N	Qualitative traits	Code	Description	Scale
1	Growth habit	GrH	Data was taken after 8 weeks of sowing based on the petiole and internode length of the middle stem of 5 middle plants per plot	(1) Accession of bunch type (P/I ≥ 9)
				(2) Accession of semi-bunch type (P/I = 7–9)
				(3) Accession of Spreading type (P/I ≤ 7)
2	Stem hairiness	StH	Data were recorded at the harvest	(1) Absent. (2) Sparse. (3) Dense
3	First stem color	FSC	Recorded after 2 weeks of planting	(1) Green (2) Reddish (3) Stripped (4) Reddish green (5) Brownish
4	Terminal leaflet shape	TLS	Data were recorded 8 weeks after sowing	(1) Round (2) Oval (3) Elliptical (4) Lanceolate
5	Terminal leaflet color	TLC	Data were recorded 8 weeks after sowing	(1) Green (2). Red (3) Purple
6	Petiole pigmentation	PetP	Data were recorded after 2 weeks of sowing	(1) Green (2) Reddish-brown (3) Brown (4) Reddish-green
7	Shape of pod	PoS	Data were taken based on 1 seeded pod at harvest	(1) Hook with ending point on the opposite side (2) Round with ending point on the opposite side (3) Hook with ending point on both sides
8	Color of pod	PoC	Data was taken using a color wheel and done at harvest	(1) Yellowish-brown (2) Reddish-brown (3) Brown (4) Cream with brown patches
9	Pod texture	PoT	Data were taken within 3–5 days of harvesting	(1) Much grooved (2) Much folded (3) Little grooved (4) Smooth
10	Seed shape	SeS	Data were taken from the seed of 1 seeded pod at harvest	(1) Oval. (2) Round (3) Other
11	Seed color	SeC	Data were taken using a color wheel and done at harvest	(1) Cream. (2) Light red (3) Light brownish red (4) Dark purple (5) Light brown (6) Purplish red
12	Eye color	EyC	Data were taken based on Bambara groundnut descriptor at harvest	(1) Absent (2) Brown (3) Light red (4) Black (5) Grey
13	Testa pattern	TeP	Data were taken based on Bambara groundnut Descriptors at harvest	(1). No testa pattern. (2) Black strips on both micropylar ends (3) Cream marbled (4) Light red strips on one micropylar end (5) Brownish red strips on both micropylar
14	Testa color with eye pattern around hilum	(TC + EyP)	Described in a combination of the testa background with eye color	(1) Light red/brown testa with light red /brown butterfly-like eye (2) No eye pattern (3) Cream testa with black/Brown butterfly like eye (4) Cream testa with grey/Light red triangular eye (5) Cream testa with light red patches (6) Purplish red testa with cream butterfly like eye

### Statistical analysis

The vegetative, yields, and phenological traits were examined using the ANOVA to determine if variations existed among the accessions and locations by making use of the R statistical packages version R-4.0.5. Fischer’s least significant difference (F-LSD) was used to separate means at a probability level of 5%. PCA was performed using the FactoMineR and factoextra packages and Pearson correlation was done using the corr. Functions in R. A hierarchical cluster analysis was performed using the ward D2 method with cluster factoextra package in R. All the R analysis commands or code used for each analysis in this study can be found as supplementary file So attached to this manuscript.

## Supplementary Information

Supplementary Information 1.

Supplementary Information 2.

Supplementary Information 3.

Supplementary Information 4.
