# Supplementary figures and images for: Genetic variation and characterization of Bambara groundnut [Vigna subterranea (L.) verdc.] accessions under multi-environments considering yield and yield components performance
Source: Sci Rep. 2023 Jan 27;13:1498. doi: 10.1038/s41598-023-28794-8 (PMC9883518; doi:10.1038/s41598-023-28794-8)

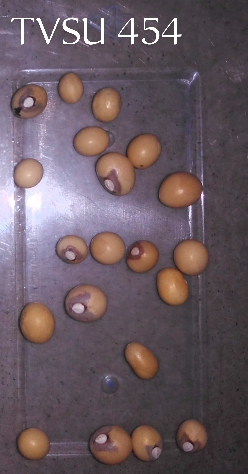

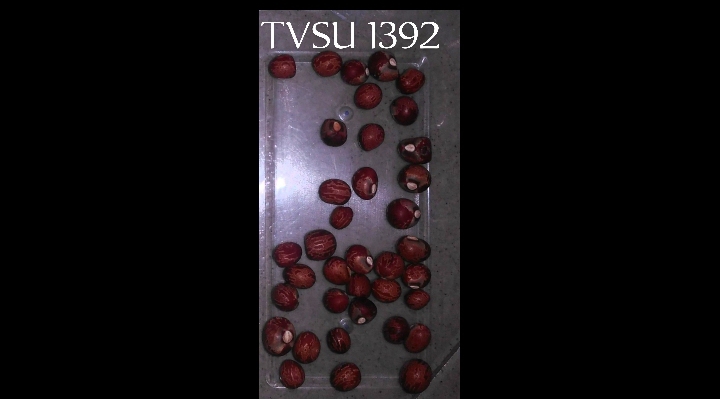


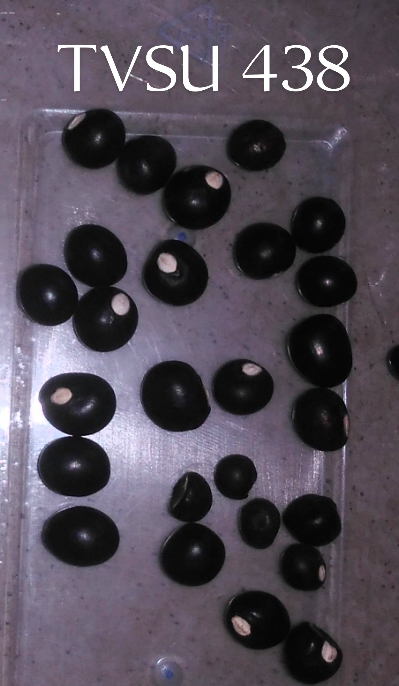

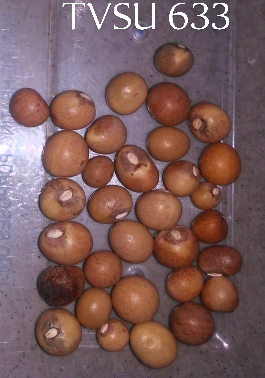


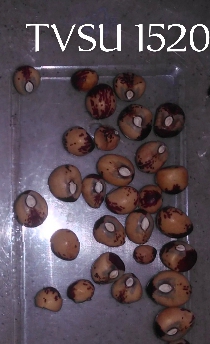

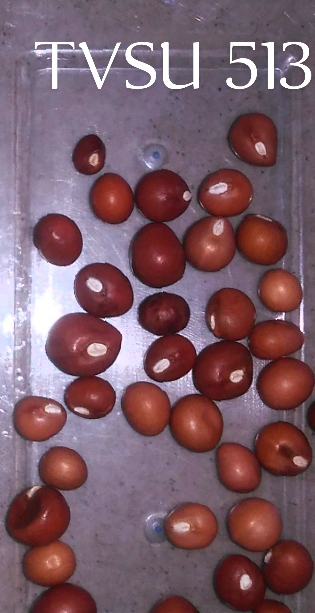


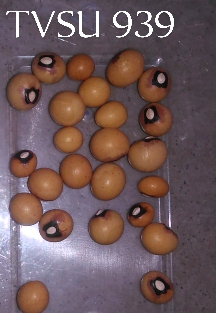

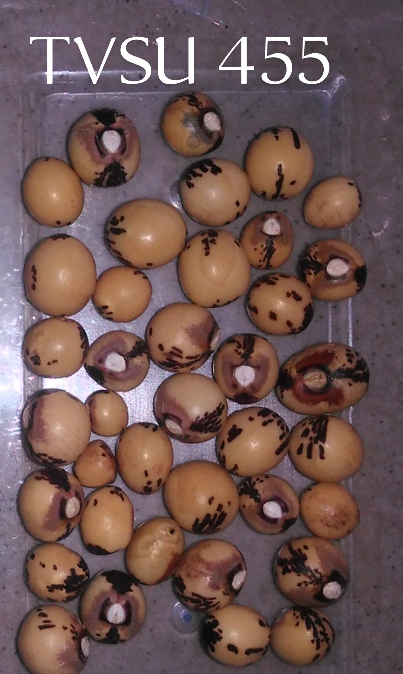


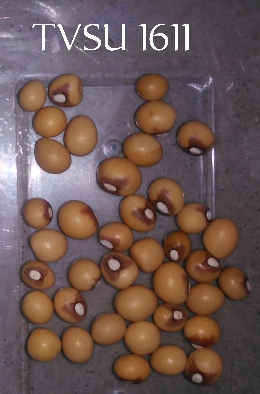

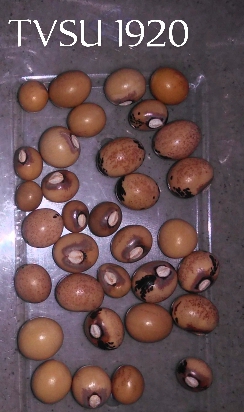


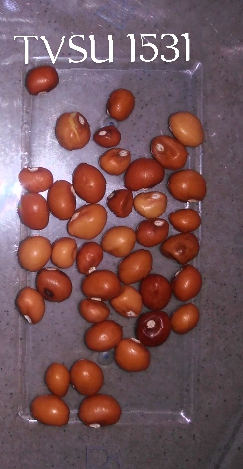

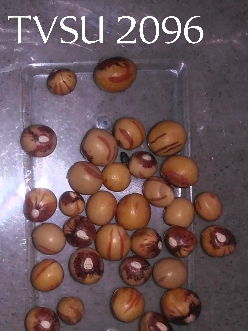


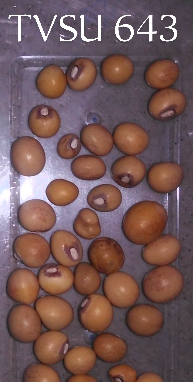

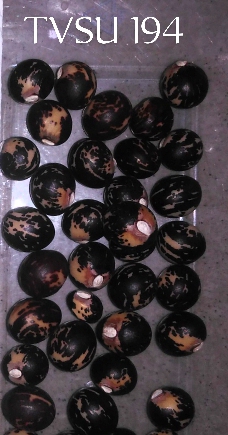


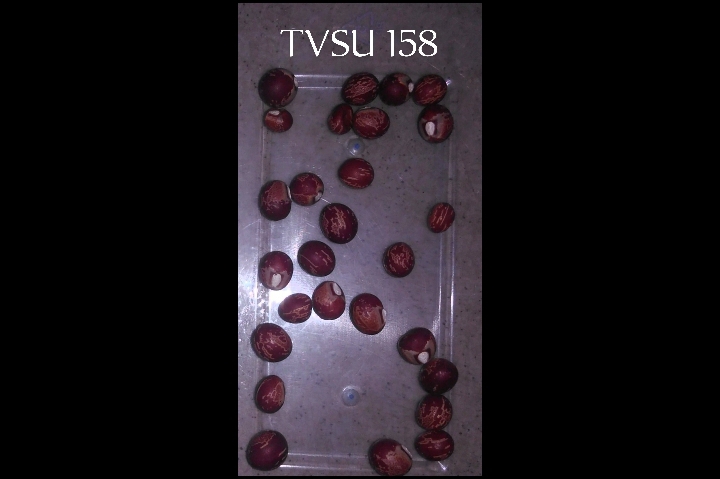

Supplement: Supplementary file 2 — Supplementary Information 2. [file 41598_2023_28794_MOESM2_ESM.docx]
