# Supplementary material for: Genetic variation and characterization of Bambara groundnut [Vigna subterranea (L.) verdc.] accessions under multi-environments considering yield and yield components performance
Source: Sci Rep. 2023 Jan 27;13:1498. doi: 10.1038/s41598-023-28794-8 (PMC9883518; doi:10.1038/s41598-023-28794-8)
Supplement: Supplementary file 3 — Supplementary Information 3. [file 41598_2023_28794_MOESM3_ESM.docx]

**Supplementary file So for R commands used**

**ANOVA: the R analysis commands or code**

getwd()

Data=read.csv("Tomato.csv")

str(Data)

Data$Block=as.factor(Data$Block)

Data$Variety=as.factor(Data$Variety)

library(agricolae)

View(Data)

model=aov(LL50~Block+Variety, Data)

summary(model)

sink(file="Outputs means separation.txt")

out=LSD.test(model,"Variety",main="LL50")

out

sink()

write.csv(file = "means separ LL50.csv", out$groups)

**PCA and Correlation: Commands**

#Import dataset

getwd() #Get working directory

Data=read.csv("Example.csv", row.names = 1)

str(Data)

View(Data)

#Interesting resources

#http://www.sthda.com/english/articles/31-principal-component-methods-in-r-practical-guide/112-pca-principal-component-analysis-essentials/

#http://www.sthda.com/english/articles/31-principal-component-methods-in-r-practical-guide/117-hcpc-hierarchical-clustering-on-principal-components-essentials/

Data = read.csv("Example.csv", row.names = 1)

str(Data) # check the nature of the variables and convert them if necessary

#####import required packages###########

library(FactoMineR)

library(factoextra)

#Run PCA with PCA function in FactoMineR

result<-PCA(Data)

head(result$var$coord) # get results for the coordinates (correlations) of the variables

res<-get_pca_var(result) # get all variables for all components whether siginificant or not.

correl=res$cor

coordin=res$coord

cntrib=res$contrib #Extract result for correlations, coordinate, contribution in your working directory

write.csv(correl, file = "Correlation with axes.csv")

write.csv(coordin, file = "Coordinates on axes.csv")

write.csv(cntrib, file = "Contribution on axes.csv")

#######plot the screeplot to cross check the selection of the components

pdf(file = "screeplot.pdf", width = 8, height = 8)

fviz_screeplot(result, addlabels = TRUE, ylim = c(0, 35))

dev.off()

#######plot the eigen values

pdf(file = "eigen values.pdf", width = 9, height = 11)

barplot(result$eig[,1],main="Eigenvalues",names.arg=1:nrow(result$eig))

dev.off()

dimdesc(result, axes=c(1,2,3,4,5)) #Summary of selected axes (here, I selected the first 5 dimensions/principal components)

plot(result, choix="ind") #Plot individuals

fviz_pca_var(result, col.var="contrib") #Plot variables with factoextra package showing contributions

fviz_pca_var(result, col.var="steelblue")+ theme_minimal() #Plot variables with factoextra package

fviz_pca_biplot(result, axes = c(1, 2), col.ind = "black", col.var = "steelblue")

tiff("Biplot.tiff", width = 6, height = 6, units = 'in', res = 300)

fviz_pca_biplot(result, axes = c(1, 2), col.ind = "black", col.var = "steelblue",

repel = TRUE, labelsize= 2) + # Avoid text overlapping

theme(text = element_text(size = 7.5))

dev.off()

##########################################################################

#Correlation analysis############

#Correlation among quantitative variables

#Load packages

library(corrgram)

library(corrplot)

library(Hmisc)

Data=read.csv("Example.csv", row.names = 1)

str(Data)

Data<-as.matrix(Data)

result<-rcorr(Data, type = "spearman") #to obtain correlation values and P values. You can use pearson or kendall depending on the nature of your data

write.csv(result$r, file = "Correlation coefficient.csv")

write.csv(result$P, file = "Pvalues.csv")

Data3<-as.matrix(result$r)

p.mat<-result$r

p.mat1<-result$p

pdf(file="Correlogram.pdf", width=11, height=8.5)

corrplot(Data3, method = c("circle"), type = c("lower"), add = FALSE,

col = NULL, bg = "white", is.corr = TRUE, diag = FALSE, outline = FALSE, addCoef.col = NULL,

addCoefasPercent = FALSE, order = c("original"), hclust.method = c("ward.D2"))

title("Correlation among quantitative variables", cex.main=1, line= -38) #to add title

dev.off()

**Hierarchical cluster analysis or Dendogram commands**

> library(cluster)

> library(factoextra)

> PCA_BAMBARA<-read.csv(file.choose(),header=TRUE)

> PCA_BAMBARA

> mydata <- scale(PCA_BAMBARA)

> fviz_nbclust(mydata, kmeans, method = "gap_stat")

> km.res <- kmeans(mydata, 3, nstart = 25)

> fviz_cluster(km.res, data = mydata, palette = "jco",

+ ggtheme = theme_minimal())

> res.hc <- hclust(dist(mydata), method = "ward.D2")

> fviz_dend(res.hc, cex = 0.5, k = 4, palette = "jco")
